# Seven new species of the spider genus *Ochyrocera* from caves in Floresta Nacional de Carajás, PA, Brazil (Araneae, Ochyroceratidae)

**DOI:** 10.3897/zookeys.726.19778

**Published:** 2018-01-10

**Authors:** Antonio D. Brescovit, Igor Cizauskas, Leandro P. Mota

**Affiliations:** 1 Laboratório Especial de Coleções Zoológicas, Instituto Butantan, Av. Vital Brasil, 1500, Butantã, São Paulo, São Paulo, Brazil, 05503-900

**Keywords:** Amazonian region, Haplogynae, taxonomy

## Abstract

Seven new species of the spider genus *Ochyrocera* from cave areas in Floresta Nacional de Carajás (state of Pará, northern Brazil) are described: *Ochyrocera varys* **sp. n.**, *Ochyrocera atlachnacha* **sp. n.**, *Ochyrocera laracna* **sp. n.**, *Ochyrocera aragogue* **sp. n.**, *Ochyrocera misspider* **sp. n.**, *Ochyrocera charlotte* **sp. n.**, and *Ochyrocera ungoliant* **sp. n.** Two groups of the species are discussed, the *quinquivittata* group that include specimens with an apparently bifid retrolateral apophysis in the cymbium of the male palp and the *arietina* group, here proposed, that include those specimens with an entire cymbium, with no retrolateral apophysis, in the male palp. Although these species were abundant inside caves, the examined specimens do not have troglomorphic characteristics and can be classified as edaphic troglophile species, capable of completing its life cycle in soil, shallow subterranean habitats, or caves.

## Introduction

Ochyroceratidae is a small spider family from the tropical areas in Neotropical, African, and Indo-Pacific regions. Its members live among litter or in caves, do not exceed 2 mm in total size, and have six eyes (Jocqué and Dippenaar-Schoeman 2006). The group currently includes 175 species distributed in 15 genera (World Spider Catalog 2017). No generic revision has been carried out to date; therefore, the family’s diversity is poorly understood.

Species from the genus *Ochyrocera* Simon, 1891 are characterized in having modifications on the cymbial apophysis, which can be triangular or conical, with an elongated base, projected forward, and often with an apical cuspule (Dupérré (2015); Pérez-González et al. 2016). The genus comprises 35 species, with the type species, *O. arietina* from Island of Saint Vincente, Antilles region (Simon 1892). From Brazil were described only six species: *Ochyrocera coerulea* Keyserling, 1891 and *O. viridissima* Brignoli, 1974, both described from the state of Santa Catarina, *Ochyrocera cornuta* Mello-Leitão, 1944 from the state of Mato Grosso, *Ochyrocera ibitipoca* Batista, González & Tourinho, 2008 from the state of Minas Gerais and *Ochyrocera hamadryas* Brignoli, 1978 from state of Amazonas (World Spider Catalog 2017).

In this paper, seven new species are described from iron caves in Floresta Nacional de Carajás, state of Pará, northern Brazil. This area has iron formations such as itabirites, ferruginous dolomites, hematite, phyllite, jaspillite, and hematite (Carmo and Jacobi 2013). Two of the new species herein described were also collected outside the caves, and none of the seven species have troglomorphic traits, being classified as edaphic troglophile specimens.

## Materials and methods

Morphological terms follow Dupérré (2015) and Pérez-González et al. (2016). Descriptions and measurements were performed using a Nikon SMZ 745T stereomicroscope. Photographs were taken with a Leica DFC 500 digital camera on a Leica MZ16A stereomicroscope. Focal range images were made using Leica Application Suite software, version 2.5.0.

The following abbreviations were used in the description:

**ALE** anterior lateral eyes;

**AME** anterior median eyes;

**PME** posterior median eyes.

Clypeus height was represented in relation to the size of the posterior median eyes (PME). Total and femur lengths were measured in lateral view without detaching any part from the specimen. All measurements are in millimeters. Female genitalia were excised with a sharp needle and photographs were taken using Hoyer´s microscope slides, following Krantz and Walter (2009). For scanning electron microscopy (SEM) images, body parts were dehydrated in a series of graded ethanol washes (80% to 100%), dried by critical point, mounted on metal stubs using adhesive copper tape and nail polish for fixation and covered with gold. SEM photographs were taken with a FEI Quanta 250 scanning electron microscope at the Laboratório de Biologia Celular of Instituto Butantan, São Paulo, Brasil.

The examined specimens are deposited in the following collections (abbreviation and curator in parentheses): Instituto Butantan, São Paulo (IBSP, A.D. Brescovit); Coleção de Invertebrados Subterrâneos da Universidade Federal de Lavras, Lavras (ISLA, R.L. Ferreira), Museu Paraense Emílio Goeldi, Belém (MPEG, A.B. Bonaldo), Museu de Zoologia da Universidade de São Paulo (MZSP, R. Pinto da Rocha).

## Taxonomy

### Ochyrocera

Taxon classificationAnimaliaAraneaeOchyroceratidae

Simon, 1892

Ochyrocera Simon, 1892: 565 (Type species, O. arietina Simon)
Ceruleocera Marples, 1955: 462 (Type species by original designation C. ransfordi Marples); Brignoli 1979: 598 (Syn.)

#### Diagnosis.

**Figure 1.**
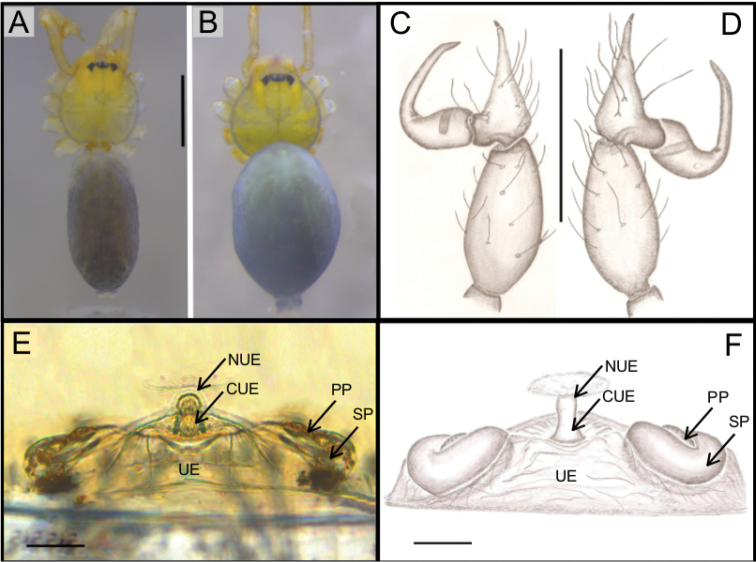
*Ochyrocera varys* sp. n., male holotype (**A, C–D**), female paratype, IBSP 176843 (**B, E–F**) **A–B** habitus, dorsal view **C** left male palp, retrolateral view **D** same, prolateral view **E** genitalia, enzyme cleared, dorsal view **F** same, dorsal view. Abbreviations: CUE = columnar uterus externus, NUE = neck of uterus externus, PP = pore-plate, SP = spermathecae, UE, uterus externus. Scale bars: 0.5 mm (**A, B**); 0.7 mm (**C, D**); 500 µm (**E, F**).

Species of the genus *Ochyrocera* can be distinguished by having a tracheal spiracle between the epigastric fold and spinnerets (see Pérez-González et al. 2016: fig. 8A); clypeus sloping (Fig. 1A–B); tip of labium notched (Fig. 8E); long legs; male palp without tibial apophysis; cymbium conical and with prolateral extension, with or without apical cuspule; and flexed embolus projecting forward (Fig. 1C–D).

#### Composition.

Thirty-nine species (World Spider Catalog 2017).

#### Distribution.

With the exception of *Ochyrocera ransfordi*, described by Marples (1955) from Samoa, all described species are from Mexico, Cuba, Hispaniola, Lesser Antilles, Saint Vincent, Guatemala, Venezuela, French Guiana, Ecuador, Peru, Argentina, and Brazil.

### Ochyrocera varys sp. n.

Taxon classificationAnimaliaAraneaeOchyroceratidae

http://zoobank.org/A3F555D5-02E7-4C30-8B5F-3C8CEE3672F7

**Figure 2.**
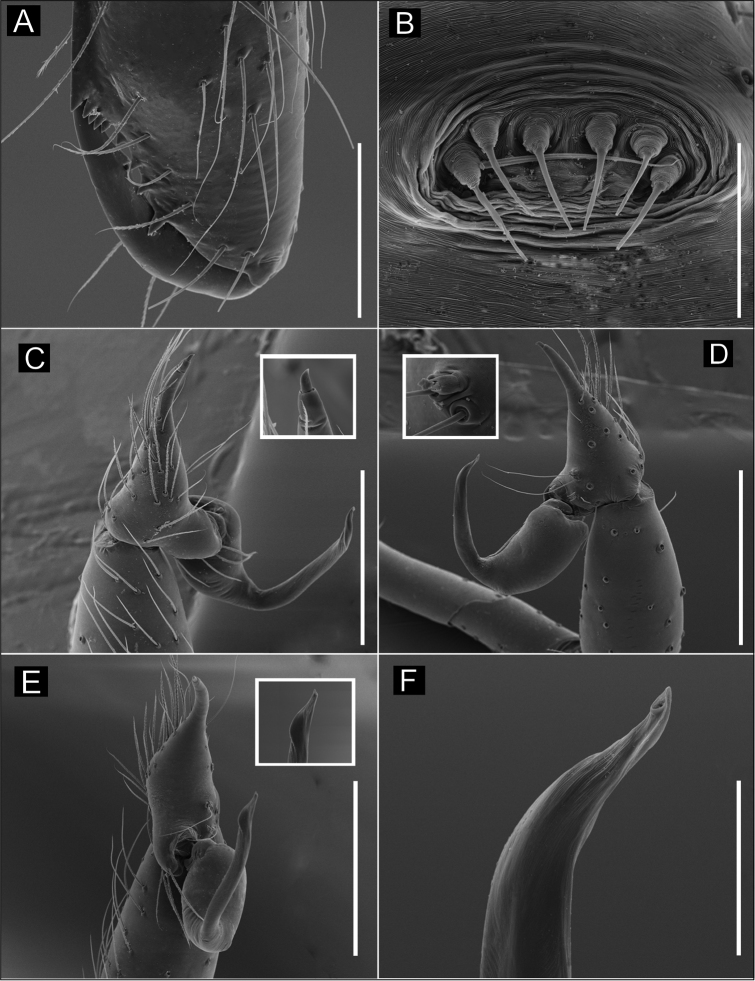
SEM images of *Ochyrocera varys* sp. n., male IBSP 174714 (**A–F**) **A** chelicerae, frontal view **B** epiandrous area, abdomen, ventral view **C–F** male palp **C** prolateral view (inset, cuspule) **D** retrolateral view (inset, base of long hair and tarsal organ) **E** ventral view (inset, embolus tip) **F** embolar tip, detail, retrolateral view. Scale bars: 100 µm.

**Figure 3.**
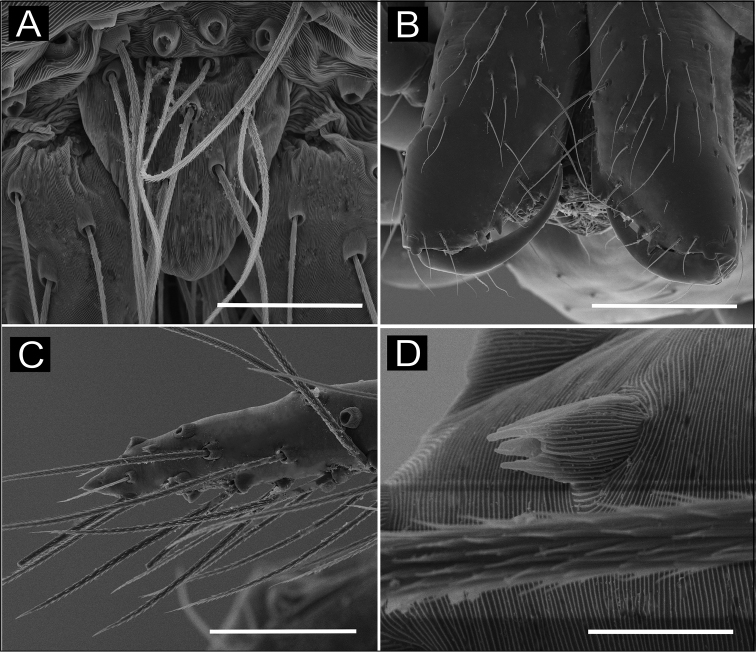
SEM images of *Ochyrocera varys* sp. n., female IBSP 174714 (**A–D**) **A** colulus, ventral view **B** chelicerae, frontal view **C** pedipalp, distal, prolateral view **D** pedipalp, tarsal organ. Scale bars: 50 µm.

Figures 1 , 2 , 3 , 19A , 21A, D

#### Types.

Holotype male from Cave N5S–0021 (6°5'15"S, 50°7'34"W), Serra Norte, Floresta Nacional de Carajás, Parauapebas, Pará, Brazil, 25/VIII–03/IX/2009, R. Andrade & I. Cizauskas (IBSP 177662). Paratypes: female from Cave N4E–0033 (6°2'25"S, 50°9'36"W), Serra Norte, Floresta Nacional de Carajás, Parauapebas, Pará, Brazil, 15–22/IX/2009, R. Andrade & I. Cizauskas (IBSP 176843); male and female from N5S–0085 (6°5'12"S, 50°7'35"W), Serra Norte, Floresta Nacional de Carajás, Parauapebas, Pará, Brazil, 25/VIII–03/IX/2009, R. Andrade & I. Cizauskas (MPEG 34434, ex IBSP 177644); 2 males, 2 females, N5S–0085 (6°5'12"S, 50°7'35"W), Serra Norte, Floresta Nacional de Carajás, Parauapebas, Pará, Brazil, 14/III–04/IV/2010, R. Andrade & I. Cizauskas (MZSP 72854, ex IBSP 177735).

#### Other material examined.

BRAZIL. Pará: **HYPOGEAN SAMPLES**: Canaã dos Carajás, Floresta Nacional de Carajás, Serra Sul, Cave CAV_0017 (6°24'23"S; 50°22'9"W), 1♀, 22–31/V/2010 (IBSP 175566); Cave CAV_0034 (6°24'9"S, 50°22'56"W), 1♂, 22–31/V/2010 (IBSP 175316); Cave CAV_0018 (6°29'50"S, 51°9'31"W), 3♀, 08–15/III/2012 (IBSP 175979) all collected by R. Andrade & I. Cizauskas et al.; Cave S11A-03 (6°20'60"S, 50°27'2"W), 2♂7♀, 23/VIII−02/IX/2007 (IBSP 174480); Cave S11A-05 (6°21'7"S, 50°27'3"W), 3♂11♀, 23/VIII−02/IX/2007 (IBSP 174405); 1♂3♀, 06/IV/2017, R. Zampaulo & X. Prous (IBSP 194762); Cave S11A-07 (6°21'6"S, 50°26'36"W), 2♂3♀, 23/VIII−02/IX/2007 (IBSP 174410); Cave S11A-12 (6°19'53"S; 50°27'4"W), 1♂5♀, 23/VIII−02/IX/2007 (IBSP 174413); Cave S11A-20 (6°19'4"S, 50°26'23"W), 1♂5♀, 23/VIII−02/IX/2007 (IBSP 174416, IBSP 174417); Cave S11A-26 (6°18'26"S, 50°26'55"W), 3♂6♀, 23/VIII-02/IX/2007 (IBSP 174423, IBSP 174418); Cave S11A-36 (6°19'1"S, 50°27'17"W), 4♂4♀, 23/VIII−02/IX/2007 (IBSP 174429); Cave S11B-09 (6°21'19"S, 50°23'24"W), 1♂1♀, 23/VIII−02/IX/2007 (IBSP 174433); Cave S11B-11 (21'27"S; 50°23'22"W), 3♀, 23/VIII−02/IX/2007 (IBSP 174435); Cave S11B-13 (6°21'16"S, 50°26'51"W), 6♀, 23/VIII−02/IX/2007 (IBSP 174439); Cave S11B-14 (6°20'59"S, 50°24'10"W), 1♀, 23/VIII−02/IX/2007 (IBSP 174441); Cave S11B-23 (6°20'43"S, 50°24'35"W), 2♀, 23/VIII−02/IX/2007 (IBSP 174443); Cave S11B-24 (6°20'43"S, 50°°24'34"W), 3♀, 23/VIII−02/IX/2007 (IBSP 174446); Cave S11B-49 (6°21'31"S, 50°23'21"W), 2♂, 23/VIII−02/IX/2007 (IBSP 174448); Cave S11C-14 (6°24'10"S, 50°22'57"W), 3♂5♀, 23/VIII−02/IX/2007 (IBSP 174451); Cave S11C-27 (6°23'51"S, 50°23'21"W), 2♀, 23/VIII−02/IX/2007 (IBSP 174453) all collected by R. Andrade & I. Arnori et al.; Cave S11D-06 (6°24'4"S, 50°21'1"W), 1♂1♀, 19–22/II/2010 (IBSP 175534); Cave S11D-11 (6°23'51"S, 50°21'30"W), 1♀, 13–30/I/2010 (IBSP 175512); Cave S11D-43 (6°24'48"S, 50°19'17"W), 1♂2♀, 13–30/I/2010 (IBSP 175521); Cave S11D-64 (6°23'31"S, 50°18'48"W), 1♂1♀, 01–14/VII/2010 (IBSP 175653); Cave S11D-78 (6°23'32"S, 50°18'58"W), 1♀, 13–30/I/2010 (IBSP 175423) all collected by R. Andrade & I. Cizauskas et al.; Parauapebas, Cave CRIS-28 (6°27'31"S, 49°42'33"W), 2♀, 29/VII−06/VIII/2008, R. Andrade (IBSP 174624); Floresta Nacional de Carajás. Serra Norte, Cave GEM-1564, 10♀, 17–24/X/2008 (IBSP 174508); Cave GEM-1590, 5♀, 17–24/X/2008, R. Andrade (IBSP 174521); Cave N1_0002 (6°2'24"S, 50°16'12"W), 1♀, 28/IX-03/X/2007, R. Andrade & I. Arnori et al. (IBSP 174652); Cave N1_0004 (6°2'23"S, 50°16'12"W), 1♂8♀, 28/IX-03/X/2007, R. Andrade & I. Arnori et al. (IBSP 174655); 3♀, 16/VII-06/VIII/2014, Equipe Carste et al. (IBSP 188836, IBSP 188842); Cave N1_0008 (6°2'20"S, 50°16'13"W), 6♀, 28/IX−03/X/2007 (IBSP 174657, IBSP 174656); Cave N1_0014 (6°2'2"S, 50°16'20"W), 4♀, 28/IX−03/X/2007 (IBSP 174662); Cave N1_0015 (6°2'2"S, 50°16'16"W), 1♂7♀, 28/IX−03/X/2007 (IBSP 174665, IBSP 174664); Cave N1_0018 (6°2'1"S, 50°16'17"W), 2♂1♀, 28/IX−03/X/2007 (IBSP 174669) all collected by R. Andrade & I. Arnori et al.; Cave N1_0019 (6°2'1"S, 50°16'17"W), 1♀, 16/VII−06/VIII/2014, Equipe Carste et al. (IBSP 188834); Cave N1_0020 (6°1'53"S, 50°18'1"W), 1♀, 28/IX−03/X/2007 (IBSP 174674); Cave N1_0022 (6°1'57"S, 50°16'19"W), 2♂ 5♀, 28/IX−03/X/2007 (IBSP 174679, IBSP 174683); Cave N1_0025 (6°1'53"S, 50°16'20"W), 6♂17♀, 28/IX-03/X/2007 (IBSP 174685) all collected by R. Andrade & I. Arnori et al.; 1♂, 02–29/IV/2015, Equipe Carste et al. (IBSP 188847); Cave N1_0037 (6°1'50"S, 50°16'28"W), 2♀, 28/IX-03/X/2007, R. Andrade & I. Arnori et al. (IBSP 174688); Cave N1_0055 (6°1'12"S, 50°16'43"W), 1♀, 16/VII−06/VIII/2014, Equipe Carste et al. (IBSP 188831); Cave N1_0064 (6°1'7"S; 50°16'45"W), 3♀, 28/IX−03/X/2007 (IBSP 174692); Cave N1_0072 (6°1'13"S; 50°17'18"W), 4♀, 28/IX−03/X/2007 (IBSP 174698) all collected by R. Andrade & I. Arnori et al.; Cave N1_0073 (6°1'13"S; 50°17'17"W), 1♂5♀, 16/VII−06/VIII/2014 (IBSP 188845, IBSP 188833, IBSP 188838); 1♀, 02–29/IV/2015, Equipe Carste et al. (IBSP 188843); Cave N1_0075 (6°1'14"S, 50°16'49"W), 2♀, 28/IX-03/X/2007 (IBSP 174699, IBSP 174700); Cave N1_0098 (6°1'9"S, 50°17'5"W), 2♀, 28/IX-03/X/2007 (IBSP 174706); Cave N1_0103 (6°0'13"S, 50°17'55"W), 3♂10♀, 28/IX-03/X/2007 (IBSP 174707, IBSP 174708) all collected by R. Andrade & I. Arnori et al.; Cave N1_0109 (6°0'41"S, 50°18'41"W), 4♂2♀, 16/VII−06/VIII/2014, Equipe Carste et al. (IBSP 188848, IBSP 188846); Cave N1_0116 (6°0'39"S, 50°18'50"W) 1♂3♀, 28/IX−03/X/2007 (IBSP 174711), collected by R. Andrade & I. Arnori et al.; Cave N1_0141 (6°2'34"S, 50°16'32"W), 1♂10♀, 16/VII-06/VIII/2014 (IBSP 188840) collected by Equipe Carste et al.; Cave N1_0143 (6°1'36"S, 50°17'27"W), 3♂8♀, 28/IX−03/X/2007 (IBSP 174718, IBSP 174714); Cave N1_0170 (6°1'23"S, 50°17'58"W), 3♀, 28/IX−03/X/2007 (IBSP 174724); Cave N1_0173 (6°1'27"S, 50°17'55"W), 3♀, 28/IX−03/X/2007 (IBSP 174726) all collected by R. Andrade & I. Arnori et al.; Cave N1–0162 (6°0'55"S, 50°18'46"W), 1♀, 02–29/IV/2015 (IBSP 188841), collected by Equipe Carste et al.; Cave N2-026 (6°3'16"S, 50°14'23"W), 1♂1♀, 26/IX-17/X/2012 (IBSP 178499); 2♂3♀, 03–17/IV/2013 (IBSP 178502, IBSP 178501); Cave N3_0003 (6°1'44"S, 50°12'3"W), 1♂, 26/IX−17/X/2012 (IBSP 178481), 1♂1♀, 05–17/III/2013 (IBSP 178504, IBSP 178505); Cave N3_0004 (6°1'45"S, 50°12'2"W), 2♂0♀, 26/IX-17/X/2012 (IBSP 178482); Cave N3_0006 (6°1'45"S, 50°12'3"W), 1♂1♀, 26/IX−17/X/2012 (IBSP 178486, IBSP 178485) 1♂1♀, 05–17/III/2013 (IBSP 178506); Cave N3_0023 (6°2'35"S, 50°13'10"W), 4♂2♀, 05–17/III/2013 (IBSP 178508, IBSP 178507) 1♂1♀, 02–23/VIII/2013 (IBSP 178539); Cave N3_0026 (6°2'39"S, 50°13'9"W), 2♂, 26/IX−17/X/2012 (IBSP 178489, IBSP 178491); 4♂1♀, 05–17/III/2013 (IBSP 178509, IBSP 178512, IBSP 178510); Cave N3_0033 (6°2'42"S, 50°13'12"W), 2♂, 26/IX−17/X/2012 (IBSP 178492); 2♂3♀, 05–17/III/2013 (IBSP 178514, IBSP 178515); Cave N3_0036 (6°2'46"S, 50°13'13"W), 1♂0♀, 26/IX−17/X/2012 (IBSP 178494); Cave N3_0037 (6°2'45"S, 50°13'14"W), 1♀, 05–17/III/2013 (IBSP 178516); 1♂1♀, 26/IX−17/X/2012 (IBSP 178495); Cave N3_0039 (6°2'24"S, 50°13'21"W), 1♂0♀, 26/IX−17/X/2012 (IBSP 178497); Cave N3_0047 (6°2'27"S, 50°13'40"W), 3♂9♀, 03–17/IV/2013 (IBSP 178527, IBSP 178531, IBSP 178564, IBSP 178529, IBSP 178525, IBSP 178532); 2♂2♀, 02–23/VIII/2013 (IBSP 178541, IBSP 178540); Cave N3_0072 (6°2'36"S, 50°13'50"W), 2♀, 03–17/IV/2013 (IBSP 178533, IBSP 178534); Cave N3_0074 (6°2'35"S; 50°13'49"W), 6♂5♀, 05–17/III/2013 (IBSP 178518, IBSP 178517, IBSP 178521, IBSP 178519, IBSP 178524); 3♂, 02–23/VIII/2013 (IBSP 178546, IBSP 178548, IBSP 178549); Cave N3_0076 (6°2'28"S; 50°13'36"W), 1♂1♀, 03–17/IV/2013 (IBSP 178537); 2♂2♀, 02–23/VIII/2013 (IBSP 178552, IBSP 178553); all collected by Equipe Carste et al.; Cave N4E_0003 (6°2'25"S; 50°9'38"W), 3♀, 20/X-01/XI/2006 (IBSP 174942); Cave N4E_0007 (6°2'21"S; 50°9'36"W), 1♀, 20/X−01/XI/2006 (IBSP 174952); Cave N4E_0008 (6°2'21"S; 50°9'36"W), 9♀, 20/X−01/XI/2006 (IBSP 174954); Cave N4E_0010 (6°2'20"S; 50°9'38"W), 1♂9♀, 20/X−01/XI/2006 (IBSP 174968); 3♂5♀, 07–12/X/2008 (IBSP 174970, IBSP 174969); 4♂7♀, 20/IV-04/V/2010 (IBSP 176878, IBSP 176877, IBSP 176879, IBSP 176881, IBSP 176880); all collected by R. Andrade & I. Cizauskas et al.; Cave N4E_0011 (6°2'20"S; 50°9'38"W), 1♂6♀, 20/X−01/XI/2006, R. Andrade et al. (IBSP 174974); 3♀, 20/IV−04/V/2010 R. Andrade & I.Cizauskas et al. (IBSP 176883, IBSP 176882); Cave N4E_0012 (6°2'16"S; 50°9'37"W), 1♂0♀, 20/X−01/XI/2006 (IBSP 174976); Cave N4E_0013 (6°2'18"S; 50°9'38"W), 1♂7♀, 20/X−01/XI/2006 (IBSP 174979); all collected by R. Andrade et al.; 8♂16♀, 20/IV−04/V/2010 (IBSP 176886, IBSP 176888, IBSP 174981, IBSP 176885, IBSP 176884, IBSP 176889, IBSP 176887, IBSP 176890, IBSP 174060), collected by R. Andrade & I.Cizauskas et al.; Cave N4E_0014 (6°2'17"S; 50°9'37"W), 3♂4♀, 20/X−01/XI/2006 (IBSP 174987) 2♂6♀, 07–12/X/2008 (IBSP 174989, IBSP 174986, IBSP 174985); all collected by R. Andrade et al.; 3♂11♀, 20/IV−04/V/2010 (IBSP 176893, IBSP 176894, IBSP 176892, IBSP 176895, IBSP 176891); all collected by R. Andrade & I. Cizauskas et al.; Cave N4E_0015 (6°2'10"S; 50°9'35"W), 1♂3♀, 20/X−01/XI/2006 (IBSP 174991, IBSP 176876); Cave N4E_0016 (6°2'6"S; 50°9'37"W), 4♀, 20/X−01/XI/2006 (IBSP 174993); all collected by R. Andrade et al.; 1♂2♀, 20/IV−04/V/2010 (IBSP 176896, IBSP 176897); Cave N4E_0019 (6°2'4"S; 50°9'37"W), 2♀, 20/X-01/XI/2006 R. Andrade et al. (IBSP 175003); 3♂8♀, 20/IV−04/V/2010, R. Andrade & I. Cizauskas et al. (IBSP 176898, IBSP 176900, IBSP 176899); Cave N4E_0021 (6°2'2"S; 50°9'37"W), 6♂5♀, 20/X−01/XI/2006, R. Andrade et al. (IBSP 175010); 3♀, 20/IV-04/V/2010 (IBSP 176901, IBSP 176902, IBSP 176905); 3♂3♀, 20/IV−04/V/2010 (IBSP 176903, IBSP 176904); all collected by R. Andrade & I. Cizauskas et al.; Cave N4E_0022 (6°2'2"S; 50°10'4"W), 3♂7♀, 20/X−01/XI/2006 (IBSP 175012); 2♂3♀, 07–12/X/2008 (IBSP 175015); collected by R. Andrade et al.; 2♂7♀, 20/IV−04/V/2010 (IBSP 176906, IBSP 176908, IBSP 176907, IBSP 174061); all collected by R. Andrade & I. Cizauskas et al.; Cave N4E_0023 (6°2'1"S; 50°10'7"W), 1♂1♀, 20/IV−04/V/2010, R. Andrade & I. Cizauskas et al. (IBSP 176909); Cave N4E_0025 (6°2'1"S; 50°10'8"W), 1♀, 20/X−01/XI/2006, R. Andrade et al. IBSP 175024); Cave N4E_0026 (6°2'14"S; 50°10'3"W), 4♂10♀, 08–12/II/2007 R. Andrade & I. Arnori et al. (IBSP 175025); 4♂10♀, 08–12/II/2007 (IBSP 17502) 2♂4♀, 07–12/X/2008 R. Andrade et al. (IBSP 175028, IBSP 175033); 2♂7♀, 18/VIII-03/IX/2009, R. Andrade & I. Cizauskas et al. (IBSP 176829, IBSP 176830, IBSP 176832, IBSP 176833, IBSP 176831); Cave N4E_0033 (6°2'25"S; 50°9'36"W) 4♂5♀, 08–12/II/2007, R. Andrade & I. Arnori et al. (IBSP 175060, IBSP 175053, IBSP 175055); 1♂7♀, 07–12/X/2008, R. Andrade & I. Cizauskas et al. (IBSP 175058, IBSP 175062, IBSP 175057); 2♂3♀, 18/VIII−03/IX/2009 (IBSP 176835) 8♂22♀, 15–22/IX/2009 (IBSP 176842, IBSP 176843, IBSP 176845, IBSP 176847, (IBSP 176849, IBSP 176836, IBSP 176846, IBSP 176844, IBSP 176848, IBSP 176837, IBSP 176841, IBSP 176838, IBSP 176839, IBSP 176840); all collected by R. Andrade & I. Cizauskas et al.; Cave N4E_0039 (6°1'58"S; 50°9'39"W) 2♀, 24–30/VII/2009 (IBSP 176850, IBSP 176851) 5♀, 19/II−04/III/2010 (IBSP 176866) collected by R. Andrade & I. Cizauskas et al.; Cave N4E_0041 (6°1'59"S; 50°9'42"W), 1♂1♀, 08–12/II/2007, R. Andrade & I. Arnori et al. (IBSP 175075); 1♂2♀, 24–30/VII/2009 R. Andrade & I. Cizauskas et al. (IBSP 176853, IBSP 176852); Cave N4E_0043 (6°1'55"S; 50°9'50"W) 1♀, 24–30/VII/2009 (IBSP 176854); Cave N4E_0044 (6°1'55"S; 50°9'50"W) 3♀, 24–30/VII/2009 (IBSP 176855, IBSP 176856) 3♀, 19/II−04/III/2010 (IBSP 176863, IBSP 176864); Cave N4E_0046 (6°2'16"S; 50°9'36"W) 1♂1♀, 19/II-04/III/2010 (IBSP 176868, IBSP 176867); Cave N4E_0051 (6°2'22"S; 50°9'38"W) 1♀, 24–30/VII/2009 (IBSP 176857); Cave N4E_0054 (6°2'1"S; 50°10'8"W) 1♂1♀, 19/II-04/III/2010 (IBSP 176865); all collected by R. Andrade & I. Cizauskas et al.; Cave N4E_0061 (6°2'21"S; 50°10'3"W) 4♂3♀, 08–12/II/2007, R. Andrade & I. Arnori et al. (IBSP 175080); 2♂9♀, 07–12/X/2008 (IBSP 175077, IBSP 175079); 2♀, 24–30/VII/2009 (IBSP 176861) 3♂7♀, 24–30/VII/2009 (IBSP 176858, IBSP 176860, IBSP 176859), all collected by R. Andrade et al.; Cave N4E_0062 (6°2'1"S; 50°9'12"W), 1♀, 24–30/VII/2009 (IBSP 176823); Cave N4E_0070 (6°1'56"S; 50°9'10"W), 1♀, 19/II−04/III/2010 (IBSP 176916); Cave N4E_0072 (6°1'56"S; 50°9'13"W), 6♀, 24–30/VII/2009 (IBSP 176826, IBSP 176827, IBSP 176828) 4♀, 19/II−04/III/2010 (IBSP 176874, IBSP 176875, IBSP 176872); Cave N4E_0080 (6°1'58"S; 50°9'4"W), 3♀, 19/II-04/III/2010 (IBSP 176869); Cave N4E_0089 (6°1'59"S; 50°9'6"W), 2♀, 24–30/VII/2009 (IBSP 176824, IBSP 176825); Cave N4WS-07 (6°5'22"S; 50°11'41"W), 1♂, 23/VIII/2010 (IBSP 176990); all collected by R. Andrade & I. Cizauskas et al.; Cave N4WS-08 (6°5'22"S; 50°11'41"W) 1♀, 07–12/X/2008, R. Andrade et al. (IBSP 174801); Cave N4WS-13 (6°3'59"S; 50°11'23"W), 2♂4♀, 20/X-01/XI/2006 (IBSP 174780); 1♂1♀, 20/IV-04/V/2010 (IBSP 176384, IBSP 176383); Cave N4WS-15 (6°3'59"S; 50°11'22"W), 14♂18♀, 20/X−01/XI/2006 (IBSP 174788, IBSP 174930, IBSP 174787, IBSP 174793, IBSP 174795, IBSP 174789), 3♂4♀, 07–12/X/2008 (IBSP 174812, IBSP 174808); all collected by R. Andrade et al.; 7♂24♀, 20/IV-04/V/2010 (IBSP 176386, IBSP 176394, IBSP 176395, IBSP 176396, IBSP 176398, IBSP 176399, IBSP 176388, IBSP 176389, IBSP 176393, IBSP 176392, IBSP 176387, IBSP 176391, IBSP 176385, IBSP 176397); all collected by R. Andrade & I. Cizauskas et al.; Cave N4WS-67 (6°4'22"S; 50°11'30"W), 1♂2♀, 18/XI−01/XII/2010 (IBSP 174070, IBSP 174069); all collected by C.R.A Souza & F.P. Franco et al.; Cave N5S-01 (6°5'27"S; 50°7'31"W), 1♀, 03/IV/2017, R. Zampaulo & X. Prous (IBSP 194763); Cave N5S-03 (6°6'18"S; 50°8'4"W) 1♂, 22/III−03/IV/2005, R. Andrade & I. Arnori et al. (IBSP 55367); 2♂1♀, 14–23/X/2009, R. Andrade & I. Cizauskas et al. (IBSP 177683, IBSP 177684, IBSP 177685); Cave N5S-05 (6°6'21"S; 50°8'1"W), 1♀, 22/III-03/IV/2005 (IBSP 55338); Cave N5S_06 (6°6'21"S; 50°8'2"W), 2♂1♀, 22/III−03/IV/2005 (IBSP 55344); Cave N5S-08 (6°6'21"S; 50°7'57"W), 1♂2♀, 22/III−03/IV/2005 (IBSP 55343); all collected by R. Andrade & I. Arnori et al.; 1♂, 07–12/X/2008 (IBSP 174524); 4♂5♀, 14–23/X/2009 (IBSP 177687, IBSP 177689, IBSP 177690, IBSP 177688); Cave N5S-09 (6°6'21"S; 50°7'53"W) 4♂12♀, 14–23/X/2009 (IBSP 177712, IBSP 177716, IBSP 177713, IBSP 177715, IBSP 177717, IBSP 177714); collected by R. Andrade & I. Cizauskas et al.; Cave N5S-10 (6°6'20"S; 50°7'53"W), 1♂, 22/III−03/IV/2005 (IBSP 55342), collected by R. Andrade & I. Arnori et al., 4♀, 07–12/X/2008 (IBSP 174526, IBSP 174528); 12♂23♀, 14–23/X/2009 (IBSP 177693, IBSP 177699, IBSP 177695, IBSP 177702, IBSP 177700, IBSP 177694, IBSP 177696, IBSP 177697, IBSP 177692, IBSP 177698, IBSP 177701, IBSP 177706); Cave N5S-11 (6°6'18"S; 50°7'47"W), 12♂25♀, 14–23/X/2009 (IBSP 177711, IBSP 177707, IBSP 177705, IBSP 177704, IBSP 177709, IBSP 177710, IBSP 177708, IBSP 177703); Cave N5S-14 (6°6'19"S; 50°8'1"W), 1♀, 14–23/X/2009 (IBSP 177691); Cave N5S-15/16 (6°6'20"S; 50°7'60"W), 1♂, 14–23/X/2009 (IBSP 177686); Cave N5S-17 (6°5'15"S; 50°7'11"W), 1♂2♀, 25/VIII−03/IX/2009 (IBSP 177667, IBSP 177668, IBSP 177669); Cave N5S-18 (6°5'11"S; 50°7'39"W), 4♀, 25/VIII-03/IX/2009 (IBSP 177632, IBSP 177672, IBSP 177673, IBSP 177674); Cave N5S-19 (6°5'13"S; 50°7'37"W), 1♀, 25/VIII-03/IX/2009 (IBSP 177671); Cave N5S-20 (6°5'15"S; 50°7'35"W), 2♂7♀, 25/VIII-03/IX/2009 (IBSP 177651, IBSP 177652, IBSP 177653, IBSP 177654, IBSP 177655); Cave N5S-21 (6°5'15"S; 50°7'34"W), 2♂10♀, 07–12/X/2008 (IBSP 174533, IBSP 174537, IBSP 174541, IBSP 174534, IBSP 174536,IBSP 174538), 5♂11♀, 25/VIII-03/IX/2009 (IBSP 177656–IBSP 177658, IBSP 177660-IBSP 177666); Cave N5S-25 (6°5'12"S; 50°7'38"W) 1♂6♀, 14–16/XII/2010 (IBSP 188854, IBSP 178176); Cave N5S-30 (6°5'18"S; 50°7'10"W), 3♂8♀, 14–16/XII/2010 (IBSP 188855–IBSP 188859 4♂8♀, 10–19/V/2011 (IBSP 188860-IBSP 188867); collected by R. Andrade & I. Cizauskas et al.; Cave N5S-37 (6°6'22"S; 50°7'57"W), 1♂6♀, 07–12/X/2008, R. Andrade & et al. (IBSP 174543, IBSP 174545, IBSP 174542); 2♂20♀, 15–21/IX/2009 (IBSP 177679, IBSP 177718, IBSP 177722, IBSP 177681, IBSP 177723, IBSP 177680, IBSP 177678); 3♂4♀, 14/III-04/IV/2010 (IBSP 177721, IBSP 177719, IBSP 177720); Cave N5S-40 (6°6'19"S; 50°8'0"W), 1♀, 15–21/IX/2009 (IBSP 177724); Cave N5S-42 (6°6'21"S; 50°8'2"W), 1♂0♀, 25/VIII−03/IX/2009 (IBSP 177659); Cave N5S-52/53 (6°6'28"S; 50°7'59"W), 6♀, 25/VIII−03/IX/2009 (IBSP 177676, IBSP 177675, IBSP 177677); 1♂5♀, 14/III-04/IV/2010 (IBSP 177633, IBSP 177725, IBSP 177726, IBSP 177634); Cave N5S-57 (6°6'31"S; 50°7'57"W), 2♂6♀, 25/VIII−03/IX/2009 (IBSP 177635, IBSP 177636); 1♂3♀, 14/III−04/IV/2010 (IBSP 177727, IBSP 177728); Cave N5S-61 (6°6'19"S; 50°8'4"W), 1♀, 15–21/IX/2009 (IBSP 177729); Cave N5S-63/64/65 (6°6'12"S; 50°8'7"W) 1♀, 15–21/IX/2009 (IBSP 177682); 2♀, 14/III-04/IV/2010 (IBSP 177730, IBSP 177731); Cave N5S-84 (6°5'13"S; 50°8'12"W) 1♀, 25/VIII−03/IX/2009 (IBSP 177650); Cave N5S-85 (6°5'12"S; 50°7'35"W), 16♂20♀, 25/VIII−03/IX/2009 (IBSP 177637–IBSP 177649, IBSP 174066), 5♂7♀, 14/III−04/IV/2010 (IBSP 177732–IBSP 177735; IBSP 174065), all collected by R. Andrade & I. Cizauskas et al.; Cave N5SM1_0008 (-6,108887; -50,134387), 2♂5♀, 31/XIII/2010 (ISLA 14614) Cave N5SM1-05 (6°6'41"S; 50°8'7"W), 2♀, 01/IX/2010 (IBSP 176989); Cave N5SM1-13 (6°6'22"S; 50°8'6"W), 1♂2♀, 29/VIII/2010 (IBSP 176992); Cave N5SM1-21 (6°6'19"S; 50°8'16"W), 4♀, 02/IX/2010 (IBSP 176993); Cave N5SM1-38 (6°6'22"S; 50°8'12"W), 1♂3♀, 25/II/2011 (IBSP 176972); Cave N5SM1-42 (6°6'26"S; 50°8'6"W), 10♀, 19/II/2011 (IBSP 176999); Cave N5SM2_0003 (6°8'31"S; 50°8'6"W), 1♂1♀, 2010–11 (ISLA 14624); Cave N5SM2_0008 (6°8'27"S; 50°8'9"W), 1♀ (ISLA 14646); Cave N5SM2_0015 (6°8'17"S; 50°8'1"W), 1♂ (ISLA 14642); Cave N5SM2_0016 (6°8'17"S; 50°7'59"W), 1♂ (ISLA 14643); Cave N5SM2_0018 (6°8'18"S; 50°8'2"W), 2♂1♀ (ISLA 14638); Cave N5SM2_0022 (6°8'8"S; 50°8'7"W), 2♀ (ISLA 14627); Cave N5SM2_0023 (-6,135119; -50,13496), 1♀ (ISLA 14639); Cave N5SM2_0024 (6°8'8"S; 50°8'6"W), 1♂1♀ (ISLA 14644); Cave N5SM2_0025 (6°8'9"S; 50°8'6"W), 1♂, (ISLA 14632); Cave N5SM2_0026 (6°8'9"S; 50°8'6"W), 3♀ (ISLA 14621); Cave N5SM2_0037 (6°7'59"S; 50°8'5"W), 1♂3♀ (ISLA 14636); Cave N5SM2_0038 (6°7'58"S; 50°8'5"W), 1♂1♀ (ISLA 14647); Cave N5SM2_0042 (6°7'57"S; 50°8'11"W), 1♂5♀ (ISLA 14635, ISLA 14648); Cave N5SM2_0043 (6°7'56"S; 50°8'10"W), 1♂1♀ (ISLA 14628); Cave N5SM2_0044 (6°7'56"S; 50°8'6"W), 1♂1♀ (ISLA 14633); Cave N5SM2_0045 (6°7'55"S; 50°8'6"W), 1♂2♀ (ISLA 14641); Cave N5SM2_0053 (6°7'49"S; 50°8'5"W), 1♂1♀ (ISLA 14629); Cave N5SM2_0054 (6°7'48"S; 50°8'4"W), 1♀ (ISLA 14637); Cave N5SM2_0056 (6°7'47"S; 50°8'5"W), 3♀ (ISLA 14640); Cave N5SM2_0057 (6°7'47"S; 50°8'5"W), 1♂3♀ (ISLA 14620); Cave N5SM2_0058 (6°7'46"S; 50°8'5"W), 1♂4♀ (ISLA 14616); Cave N5SM2_0064 (6°7'43"S; 50°8'7"W), 1♀ (ISLA 14625); Cave N5SM2_0074 (6°7'32"S; 50°7'56"W), 2♂7♀ (ISLA 14623); Cave N5SM2_0076 (6°7'31"S; 50°7'54"W), 1♀ (ISLA 14645); Cave N5SM2_0078 (6°7'23"S; 50°7'49"W), 3♂5♀ (ISLA 14630, ISLA 14631); Cave N5SM2_0086 (6°7'16"S; 50°7'47"W), 1♂6♀ (ISLA 14618); Cave N5SM2_0087 (6°7'16"S; 50°7'43"W), 1♂1♀ (ISLA 14626); Cave N5SM2_0093 (6°7'17"S; 50°7'56"W), 6♂8♀ (ISLA 14619); Cave N5SM2_0096 (6°8'6"S; 50°8'12"W), 1♂1♀ (ISLA 14634), 2010–2011, all collected by Equipe UFLA; Cave N5W-03 (6°4'53"S; 50°8'4"W), 6♂2♀, 02–23/VIII/2013 (IBSP 178554, IBSP 178557, IBSP 178559, IBSP 178560, IBSP 178555, IBSP 178558) ; Cave N8-0024 (6°10'24"S; 50°9'8"W), 2♂5♀, 16/VII-06/VIII/2014 (IBSP 188830, IBSP 188832, IBSP 188835, IBSP 188839, IBSP 188844), 1♂, 02–29/IV/2015 (IBSP 188849); Cave N8-0026 (6°10'14"S; 50°9'28"W), 1♀, 16/VII−06/VIII/2014 (IBSP 188837), all collected by Equipe Carste et al.; Cave N5E–006 (6°05'05"S, 50°07'50"W), 1♂ 1♀, 22/III–03/V/2005, R. Andrade & I. Armoni (IBSP 55344); Cave N5E_0008 (6°04'54"S, 50°07'50"W), 1♂ 2♀, 22/III–03/V/2005, R. Andrade & I. Arnoni (IBSP 55343); Cave N5E_0005 (6°05'10"S, 50°07'48"W), 22/III–03/V/2005, 1♂ 1♀, R. Andrade & I. Arnoni (IBSP 55338); Cave N5E_0001 6°04'25"S, 50°07'05"W), 1♂, 22/III–03/V/2005, R. Andrade & I. Arnoni (IBSP 55342); **EPIGEAN SAMPLES**: Parauapebas, Floresta Nacional de Carajás, Serra Norte (6°5'19"S; 50°7'13"W), 2♀, 25/IV−03/V/2012 (IBSP 191339, IBSP 191357); (6°5'31"S; 50°7'35"W), 1♀, 25/IV−03/V/2012 (IBSP 191351); (6°6'12"S; 50°7'54"W), 7♂7♀, 27/I/2012 (IBSP 191340–IBSP 191348); (6°6'8"S; 50°7'54"W), 1♀, 26/IV−03/V/2012 (IBSP 191349); (6°6'17"S; 50°7'48"W), 1♂2♀, 26/IV−03/V/2012 (IBSP 191381, IBSP 191350), all collected by I. Cizauskas & R. Andrade et al.; (6°3'2"S; 50°14'56"W), 1♂, 10/X/2012 (IBSP 191393); (6°2'33"S; 50°13'6"W), 1♀, 28/IX/2012 (IBSP 191394); (6°3'9"S; 50°14'31"W), 1♀, 14/III/2013 (IBSP 191395), all collected by Equipe Carste et al.

#### Etymology.

The specific name refers to Varys, a fictional character in George R. R. Martin’s book, “A Song of Ice and Fire”. Lorde Varys is a character with a venomous spirit, known as a spider in the plot.

#### Diagnosis.

**Figure 4.**
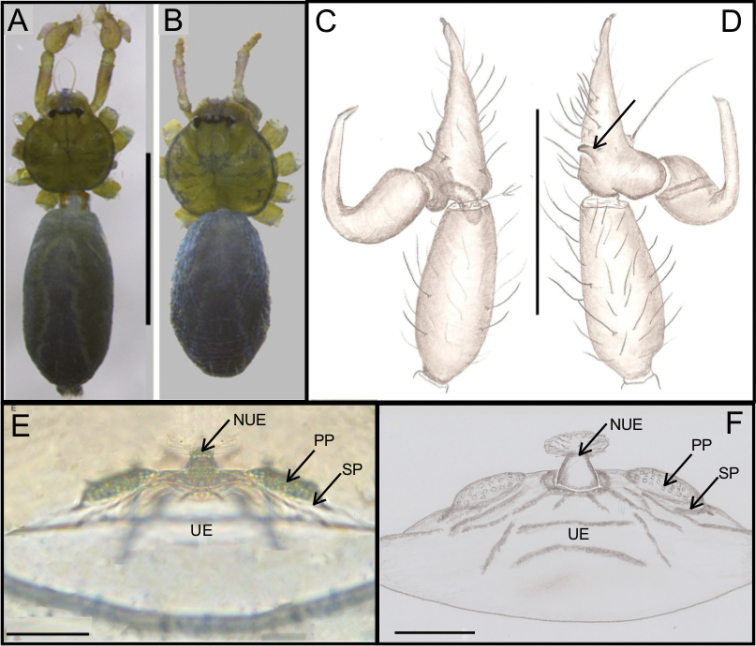
*Ochyrocera atlachnacha* sp. n., male holotype (**A, C, D**), female paratype, IBSP 188899 (**B, E, F**) **A–B** habitus, dorsal view **C** left male palp, retrolateral view **D** same, prolateral view (arrow = cymbial lateral projection) **E** genitalia, enzyme cleared, dorsal view **F** same, dorsal view. Abbreviations: CUE = columnar uterus externus, NUE = neck of uterus externus, PP = pore-plate, SP = spermathecae, UE, uterus externus. Scale bars: 0.5 mm (**A, B**); 0.7 mm (**C, D**); 500 µm (**E, F**).

*Ochyrocera varys* resembles *Ochyrocera atlachnacha* in its carapace, which is yellow and bright lime (Figs 1A–B; 4A–B) and palp with conical, elongated cymbial apophysis, and have a distal cuspule on the cymbial apophysis (Figs 1C–D, 4C–D). This species can be distinguished by the male palp having a cymbial apophysis without an accentuated lateral projection (present in *O. atlachnacha*) and by the curved distal area of embolus (Figs 1C–D, 2C–F); females have a thick spermathecae enveloping large pore-plates (Fig. 1E–F).

#### Description.

**Male** (holotype). Total length 2.3. Carapace length 0.7, ovoid; narrowing gradually anteriorly with yellow and bright lime pattern, fovea flattened and inconspicuous (Fig. 1A). Clypeus length 0.7. Eyes: PME oval; ALE and PLE rounded. Chelicerae light yellow; promargin with eight teeth, attached to long lamina (Fig. 2A); retromargin without teeth. Sternum yellow. Endites yellowish. Legs: light yellow; formula 1423; total length: I 7.0; II 5.9; III 4.1; IV 6.5. Male palp: palpal femur length 0.4; palpal tibia enlarged basally with several trichobothria; cymbial apophysis slightly curved distally, short apical cuspule, retrolateral paired long hair on non-projected base, a single tarsal organ, with two basal setae (Fig. 2C–D), cymbial prolateral extension subtriangular (Fig. 2C); bulb oval; embolus elongated, wide at base and projecting upward, with sinuous tip (Fig. 2C–F). Abdomen length 1.3, oval; uniformly grayish-green; six epiandrous spigots, with short base (Fig. 2B).

**Female** (paratype IBSP 176843). Total length: 2.0; carapace length: 0.74; Carapace pattern as in male (Fig. 1B). Pedipalp without claw, with conical tip and subdistal tarsal organ (Fig. 3C–D). Clypeus: 0.67 diameter; Eyes, chelicerae, sternum, endites and labium as in male (Fig. 3B). Legs as in male; formula 4123, total length: I 6.3; II 4.7; III 3.6 IV4.3. Abdomen length 0.97. Colulus triangular with long bristles (Fig. 3A). Internal genitalia with well-developed medial columnar uterus externus, shorter than spermathecae length and internally with inconspicuous chambers. Uterus externus ending in a narrow neck. Rounded pore-plates covered by spermathecae, with approximately 15 glandular ducts (Fig. 1E–F).

#### Distribution.

Recorded from caves and epigean areas of Carajás, state of Pará, northern Brazil (Fig. 19A).

### Ochyrocera atlachnacha sp. n.

Taxon classificationAnimaliaAraneaeOchyroceratidae

http://zoobank.org/BA66C49B-BA97-4B0E-8C18-5EBB21D0EB1C

**Figure 5.**
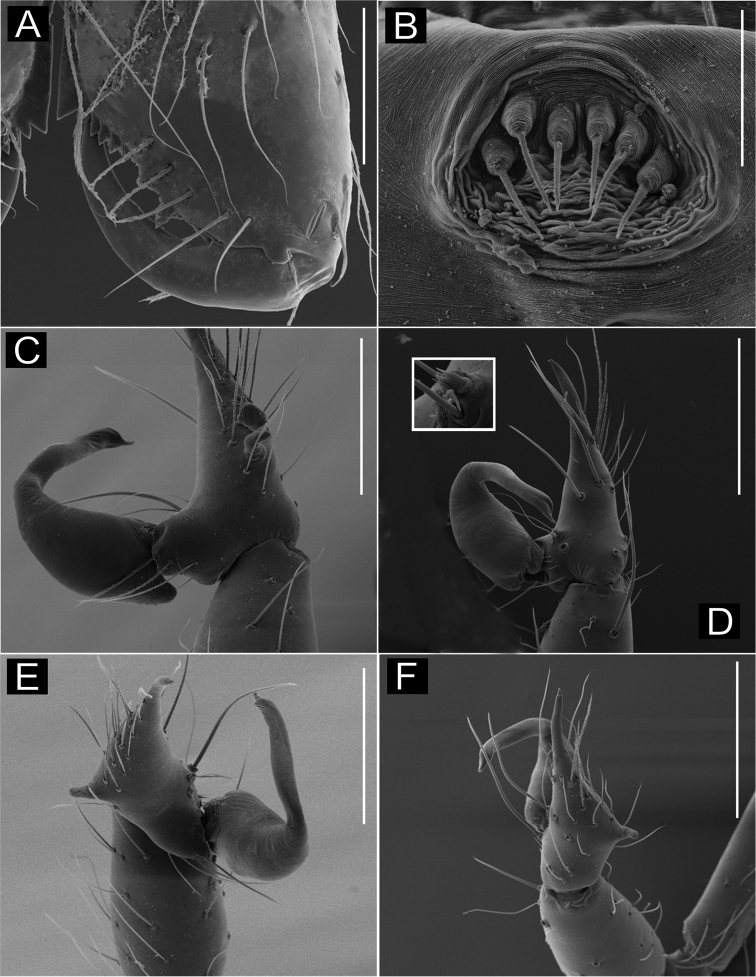
SEM images of *Ochyrocera atlachnacha* sp. n. male IBSP 175516 (**A–F**) **A** chelicerae, frontal view, **B** epiandrous, abdomen, ventral view **C–F** male palp **C** prolateral view **D** retrolateral view (inset, base of long hair and tarsal organ) **E** subapical view **F** dorsal view. Scale bars: 0.3 mm.

**Figure 6.**
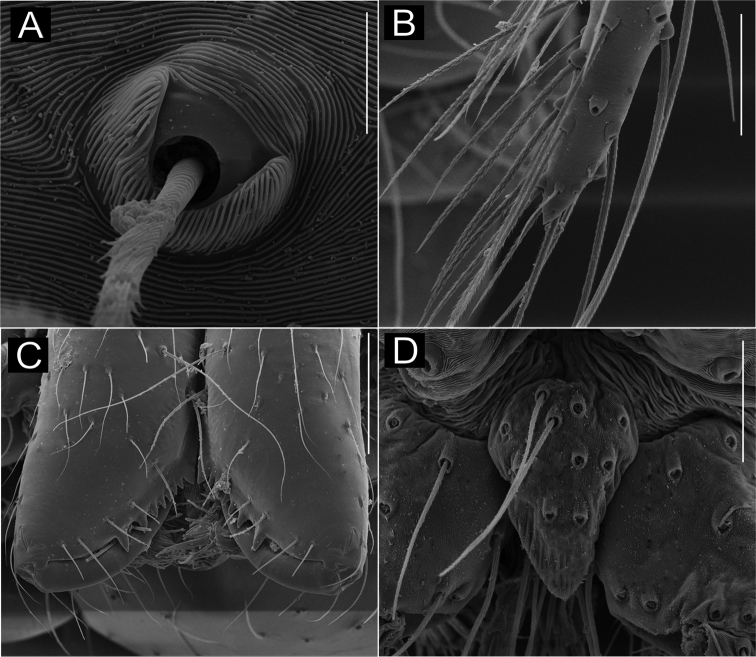
SEM images of *Ochyrocera atlachnacha* sp. n., female IBSP 175516 (**A–D**) **A** trichobothria, dorsal view **B** pedipalp, distal, prolateral view **C** chelicerae, frontal view **D** colulus, ventral view. Scale bars: 0.3 mm.

Figures 4 , 5 , 6 , 19B , 21B

#### Types.

Male holotype from Cave CAV_0034 (50°22'56"W, 6°24'9"S), Serra Sul, Floresta Nacional de Carajás, Canaã dos Carajás, Pará, Brazil, 22–31/V/2010, R. Andrade & I. Cizauskas et al., deposited in IBSP 175570. Paratypes: female from Cave S11D-64 (50°18'48"W, 6°23'31"S), Serra Sul, Floresta Nacional de Carajás, Canaã dos Carajás, Pará, Brazil, 13–30/I/2010, R. Andrade & I. Cizauskas et al. (IBSP 188899); 2♂ 2♀ from Cave S11D-64, as above (MPEG 34435, ex IBSP 175494); 1♂ 1♀ from S11-0011 (6°26'11"S; 50°17'40"W), Serra Sul, Floresta Nacional de Carajás, Canaã dos Carajás, Pará, Brazil, 03–19/VIII/2010, R. Andrade & I. Cizauskas et al. (MZSP 72855, ex IBSP 175664).

#### Other material examined.

BRAZIL. Pará: **HYPOGEAN SAMPLES**: Canaã dos Carajás, Floresta Nacional de Carajás, Serra Sul, Cave CAV_0001 (6°24'42"S; 50°20'5"W), 1♂2♀, 22–31/V/2010 (IBSP 175562, IBSP 175563); 1♂, 22–28/IX/2010 (IBSP 175659); Cave CAV_0003 (6°24'41"S; 50°20'5"W), 2♀, 22–31/V/2010 (IBSP 175564, IBSP 175565); Cave CAV_0006 (6°24'40"S; 50°19'57"W), 1♂, 22–28/IX/2010 (IBSP 175660); Cave CAV_0018 (6°24'23"S; 50°22'9"W), 1♀, 22–31/V/2010 (IBSP 175567); Cave CAV_0020 (6°24'21"S; 50°22'8"W), 1♀, 22–28/IX/2010 (IBSP 175661) 1♂, 22–31/V/2010 (IBSP 175568); Cave CAV_0021 (6°24'20"S; 50°22'10"W), 1♀, 22–31/V/2010 (IBSP 175569); 1♂2♀, 22–28/IX/2010 (IBSP 175663, IBSP 175662); Cave CAV_0034 (6°24'9"S; 50°22'56"W), 1♀, 17/X–23/X/2014 (IBSP 188882); Cave CAV_0034 (6°24'9"S; 50°22'56"W), 3♂6♀, 22–31/V/2010 (IBSP 175573, IBSP 175574,IBSP 175575, IBSP 175570, IBSP 175571, IBSP 175572) 1♂1♀, 17/X–23/X/2014 (IBSP 188881); Cave S11–03 (6°26'12"S; 50°17'38"W), 1♂, 19–22/III/2010 (IBSP 175556); Cave S11–05 (6°26'19"S; 50°17'35"W), 1♀, 19–22/III/2010 (IBSP 175557); Cave S11–11 (6°26'11"S; 50°17'40"W), 1♀, 24/II–04/III/2010 (IBSP 175555); 2♂2♀, 03–19/VIII/2010 (IBSP 175664); Cave S11–19 (6°26'36"S; 50°17'30"W) 3♀, 19–22/III/2010 (IBSP 175558, IBSP 175559); Cave S11–20 (6°26'37"S; 50°17'30"W), 1♀, 03–19/VIII/2010 (IBSP 175665); 1♀, 19–22/III/2010 (IBSP 175560); Cave S11–21 (6°26'40"S; 50°17'28"W), 1♀, 19–22/III/2010 (IBSP 175561); Cave S11D–01 (6°23'54"S; 50°21'25"W), 4♀, 13–30/I/2010 (IBSP 175462, IBSP 175461); 3♂4♀, 17/X–23/X/2014 (IBSP 188889, IBSP 188884, IBSP 188885, IBSP 188886); 3♂1♀, 01–14/VII/2010 (IBSP 175595, IBSP 175596, IBSP 175594); Cave S11D–05 (6°24'3"S; 50°20'60"W), 1♂3♀, 01–14/VII/2010 (IBSP 175597, IBSP 175598) 1♂1♀, 19–22/II/2010 (IBSP 175523, IBSP 175524); Cave S11D–06 (6°24'4"S; 50°21'1"W), 1♂1♀, 01–14/VII/2010 (IBSP 175599, IBSP 175600); 3♂6♀, 19–22/II/2010 (IBSP 175532, IBSP 175533, IBSP 175535, IBSP 175536), all collected by R. Andrade & I. Cizauskas et al.; Cave S11D–10 (6°23'54"S; 50°21'25"W), 3♀, 23/VIII–02/IX/2007, R. Andrade & I. Arnori et al. (IBSP 174462); 2♂2♀, 01–14/VII/2010 (IBSP 175601, IBSP 175602, IBSP 175603) 2♀, 13–30/I/2010 (IBSP 175443, IBSP 175444) 1♂2♀, 17/X–23/X/2014 (IBSP 188888, IBSP 188891, IBSP 188893); Cave S11D–104 (6°23'50"S; 50°21'59"W), 5♀, 14–19/XII/2011 (IBSP 175884); 1♂2♀, 30/VII–02/IX/2011 (IBSP 175883); Cave S11D–108 (6°23'58"S; 50°21'18"W), 1♂1♀, 14–19/XII/2011 (IBSP 175885); Cave S11D–11 (6°23'51"S; 50°21'30"W), 2♀, 17/X–23/X/2014 (IBSP 188887, IBSP 188890); Cave S11D–110 (6°23'49"S; 50°20'27"W), 1♂, 30/VII–02/IX/2011 (IBSP 175879); Cave S11D–111 (6°23'48"S; 50°20'27"W), 1♂3♀, 30/VII–02/IX/2011 (IBSP 175881, IBSP 175880), 2♂3♀, 14–19/XII/2011 (IBSP 175886, IBSP 175887); Cave S11D–112 (6°24'46"S; 50°21'14"W), 1♀, 30/VII–02/IX/2011 (IBSP 175882); 1♂3♀, 14–19/XII/2011 (IBSP 175888); Cave S11D–114 (6°25'15"S; 50°18'53"W), 2♂, 14–19/XII/2011 (IBSP 175889, IBSP 175890); Cave S11D–12 (6°23'46"S; 50°21'34"W), 3♀, 01–14/VII/2010 (IBSP 175604, IBSP 175608, IBSP 175609), all collected by R. Andrade & I. Cizauskas et al.; Cave S11D–12 (6°23'46"S; 50°21'34"W), 2♀, 23/VIII–02/IX/2007, R. Andrade & I. Arnori et al. (IBSP 174465, IBSP 174466); 3♂30♀, 13–30/I/2010 (IBSP 175445, IBSP 175446, IBSP 175454, IBSP 175456, IBSP 175458, IBSP 175459, IBSP 175447, IBSP 175455, IBSP 175448, IBSP 175450, IBSP 175451, IBSP 175452, IBSP 175449, IBSP 175453, IBSP 175457); 1♀, 17/X–23/X/2014 (IBSP 188892) 1♂9♀, 01–14/VII/2010 (IBSP 175605, IBSP 175607, IBSP 175606); Cave S11D–13 (6°24'6"S; 50°21'8"W), 1♂9♀, 13–30/I/2010 (IBSP 175499, IBSP 175500, IBSP 175501, IBSP 175502); 4♀, 01–14/VII/2010 (IBSP 175610, IBSP 175611, IBSP 175612); Cave S11D–14 (6°24'6"S; 50°21'10"W), 2♂4♀, 13–30/I/2010 (IBSP 175473, IBSP 175474, IBSP 175475, IBSP 175476); 2♂3♀, 01–14/VII/2010 (IBSP 175614, IBSP 175615, IBSP 175616); Cave S11D–15 (6°23'45"S; 50°21'25"W), 1♂1♀, 01–14/VII/2010 (IBSP 175618, IBSP 175617); Cave S11D–16 (6°23'47"S; 50°21'37"W), 1♂, 13–30/I/2010 (IBSP 175471); Cave S11D–17 (6°23'56"S; 50°21'23"W), 2♂1♀, 13–30/I/2010 (IBSP 175441, IBSP 175442); 3♀, 01–14/VII/2010 (IBSP 175619, IBSP 178177); Cave S11D–22 (6°24'46"S; 50°21'33"W), 3♂4♀, 19–22/II/2010 (IBSP 175541, IBSP 175542, IBSP 175543); 1♀, 03–19/VIII/2010 (IBSP 175620); Cave S11D–23 (6°24'46"S; 50°21'32"W), 1♀, 19–22/II/2010 (IBSP 175540); Cave S11D–24 (6°24'46"S; 50°21'15"W), 1♂2♀, 19–22/II/2010 (IBSP 175537, IBSP 175538, IBSP 175539); Cave S11D–26 (6°24'49"S; 50°21'17"W), 2♂1♀, 19–22/II/2010 (IBSP 175544, IBSP 175545); 1♂3♀, 03–19/VIII/2010 (IBSP 175621, IBSP 175622); Cave S11D–27 (6°24'43"S; 50°21'9"W); 1♂, 19–22/II/2010 (IBSP 175546), 2♂1♀, 03–19/VIII/2010 (IBSP 175623, IBSP 175624); Cave S11D–28 (6°24'40"S; 50°21'6"W), 1♀, 03–19/VIII/2010 (IBSP 175625); Cave S11D–29 (6°24'40"S; 50°20'44"W), 2♂5♀, 19–22/II/2010 (IBSP 175547, IBSP 175548, IBSP 175549, IBSP 175550); 1♀, 03–19/VIII/2010 (IBSP 175626); Cave S11D–31 (6°24'41"S; 50°20'43"W) 2♀, 19–22/II/2010 (IBSP 175551, IBSP 175552); Cave S11D–32 (6°24'40"S; 50°20'38"W), 2♀, 13–30/I/2010 (IBSP 175460), all collected by R. Andrade & I. Cizauskas et al.; Cave S11D–33 (6°24'40"S; 50°20'37"W), 4♂5♀, 23/VIII–02/IX/2007 (IBSP 174467); collected by R. Andrade & I. Arnori et al.; 3♂4♀, 13–30/I/2010 (IBSP 175506, IBSP 175507, IBSP 175508, IBSP 175509, IBSP 175510, IBSP 175511); 3♂6♀, 03–19/VIII/2010 (IBSP 175627, IBSP 175628, IBSP 175629, IBSP 175630, IBSP 175631); Cave S11D–34 (6°24'41"S; 50°20'36"W), 1♀, 13–30/I/2010 (IBSP 175515); Cave S11D–35 (6°24'40"S; 50°20'35"W), 1♂1♀, 13–30/I/2010 (IBSP 175517, IBSP 175518); 2♂1♀, 03–19/VIII/2010 (IBSP 174072, IBSP 175632); 2♂1♀, 17/X–23/X/2014 (IBSP 188883, IBSP 188894); Cave S11D–36 (6°24'40"S; 50°20'34"W), 1♀, 13–30/I/2010 (IBSP 175513); Cave S11D–37 (6°24'46"S; 50°21'31"W), 2♀, 13–30/I/2010 (IBSP 175553, IBSP 175554), all collected by R. Andrade & I. Cizauskas et al.; Cave S11D–39 (6°23'46"S; 50°20'27"W), 1♂5♀, 23/VIII–02/IX/2007 (IBSP 174472, IBSP 174473, IBSP 174471), R. Andrade & I. Arnori et al.; 2♀, 03–19/VIII/2010 (IBSP 175633, IBSP 175634); 6♀, 13–30/I/2010 (IBSP 175525, IBSP 175527, IBSP 175528, IBSP 175529, IBSP 175530, IBSP 175531); 1♂, 13–30/I/2010 (IBSP 175526); 03–19/VIII/2010 (IBSP 175635); all collected by R. Andrade & I. Cizauskas et al.; Cave S11D–40 (6°24'38"S; 50°19'29"W), 1♀, 23/VIII–02/IX/2007 (IBSP 174476); collected by R. Andrade & I. Arnori et al.; 1♂1♀, 13–30/I/2010 (IBSP 175516); 1♂4♀, 03–19/VIII/2010 (IBSP 175636, IBSP 175637, IBSP 175638); all collected by R. Andrade & I. Cizauskas et al.; Cave S11D–41 (6°24'38"S; 50°19'29"W), 1♂1♀, 13–30/I/2010 (IBSP 175472) 1♀, 03–19/VIII/2010 (IBSP 175639); Cave S11D–43 (6°24'48"S; 50°19'17"W), 1♀, 23/VIII–02/IX/2007 (IBSP 174478) R. Andrade & I. Arnori et al., 2♂4♀, 03–19/VIII/2010 (IBSP 175641, IBSP 175520, IBSP 175519, IBSP 175522), 2♂2♀, 03–19/VIII/2010 (IBSP 175640); Cave S11D–46 (6°24'53"S; 50°18'59"W), 2♀, 13–30/I/2010 (IBSP 175514); 3♀, 03–19/VIII/2010 (IBSP 175642, IBSP 175643); Cave S11D–50 (6°24'25"S; 50°19'14"W), 1♀, 13–30/I/2010 (IBSP 175470); 1♀, 01–14/VII/2010 (IBSP 175644); Cave S11D–51 (6°24'25"S; 50°19'14"W), 2♀, 13–30/I/2010 (IBSP 175463); Cave S11D–52 (6°24'25"S; 50°19'15"W), 1♀, 13–30/I/2010 (IBSP 175464); Cave S11D–53 (6°24'25"S; 50°19'15"W), 5♂7♀, 13–30/I/2010 (IBSP 175465, IBSP 175466, IBSP 175467, IBSP 175468); 1♂4♀, 01–14/VII/2010 (IBSP 175645, IBSP 175646, IBSP 175647); all collected by R. Andrade & I. Cizauskas et al.; Cave S11D–55 (6°24'23"S; 50°19'12"W), 1♂3♀, 23/VIII–02/IX/2007 (IBSP 174485, IBSP 174487); R. Andrade & I. Arnori et al.; 3♂3♀, 13–30/I/2010 (IBSP 175479, IBSP 175480, IBSP 175481, IBSP 175482); 3♀, 01–14/VII/2010 (IBSP 175648, IBSP 175649); Cave S11D–56 (6°24'24"S; 50°19'12"W), 2♀, 13–30/I/2010 (IBSP 175469); Cave S11D–58 (6°24'21"S; 50°19'13"W), 1♀, 13–30/I/2010 (IBSP 175477); 1♀, 01–14/VII/2010 (IBSP 175650); Cave S11D–61 (6°23'33"S; 50°18'47"W), 1♂4♀, 13–30/I/2010 (IBSP 175503, IBSP 175504, IBSP 175505); 1♀, 01–14/VII/2010 (IBSP 175651); Cave S11D–62 (6°23'32"S; 50°18'47"W), 1♂, 13–30/I/2010 (IBSP 175496) all collected by R. Andrade & I. Cizauskas et al.; Cave S11D–64 (6°23'31"S; 50°18'48"W), 1♂7♀, 23/VIII–02/IX/2007, R. Andrade & I. Arnori et al. (IBSP 174489); 13♂28♀, 13–30/I/2010 (IBSP 175484, IBSP 175493, IBSP 175495, IBSP 175492, IBSP 175486, IBSP 175491, IBSP 175489, IBSP 175494, IBSP 175485, IBSP 175483, IBSP 175487, IBSP 175490) 3♂4♀, 01–14/VII/2010 (IBSP 175655, IBSP 175657, IBSP 175658, IBSP 175652, IBSP 175654, IBSP 175656); Cave S11D–66 (6°23'34"S; 50°18'53"W), 1♀, 13–30/I/2010 (IBSP 175498); Cave S11D–67 (6°23'34"S; 50°18'53"W), 1♀, 13–30/I/2010 (IBSP 175497); Cave S11D–71 (6°23'34"S; 50°19'8"W), 1♀, 13–30/I/2010 (IBSP 175418); Cave S11D–77 (6°23'33"S; 50°18'59"W), 2♀, 13–30/I/2010 (IBSP 175422); 1♂8♀, 01–14/VII/2010 (IBSP 175576, IBSP 175577, IBSP 175579, IBSP 175580, IBSP 175578); all collected by R. Andrade & I. Cizauskas et al.; Cave S11D–78 (6°23'32"S; 50°18'58"W) 1♀, 23/VIII–02/IX/2007 R. Andrade & I. Arnori et al., (IBSP 174490); 1♂5♀, 13–30/I/2010 (IBSP 175425, IBSP 175427, IBSP 175424); 1♀, 13–30/I/2010 (IBSP 175426); 1♀, 01–14/VII/2010 (IBSP 175581); Cave S11D–79 (6°23'33"S; 50°18'56"W), 1♀, 13–30/I/2010 (IBSP 175437) 3♀, 01–14/VII/2010 (IBSP 175582, IBSP 175583); Cave S11D–80 (6°23'34"S; 50°18'57"W), 1♀, 13–30/I/2010 (IBSP 175433); 2♀, 01–14/VII/2010 (IBSP 175584); Cave S11D–81 (6°23'34"S; 50°18'53"W); 2♂7♀, 13–30/I/2010 (, IBSP 175428, IBSP 175429, IBSP 175430, IBSP 175431, IBSP 175432) 3♂8♀, 01–14/VII/2010 (IBSP 175585, IBSP 175587, IBSP 175586, IBSP 175588, IBSP 175589); Cave S11D–83 (6°23'48"S; 50°19'25"W), 1♀, 13–30/I/2010 (IBSP 175438); Cave S11D–86 (6°23'47"S; 50°19'24"W), 1♂, 13–30/I/2010 (IBSP 175439); 1♂1♀, 01–14/VII/2010 (IBSP 175590); Cave S11D–88 (6°23'45"S; 50°19'22"W), 3♀, 13–30/I/2010 (IBSP 175434, IBSP 175435, IBSP 175436); Cave S11D–94 (6°23'40"S; 50°19'17"W), 3♀, 13–30/I/2010 (IBSP 175419, IBSP 175420, IBSP 175421), all collected by R. Andrade & I. Cizauskas et al.; Cave S11D–96 (6°23'37"S; 50°19'27"W), 3♀, 23/VIII–02/IX/2007 (IBSP 174498) R. Andrade & I. Arnori et al., 1♂5♀, 13–30/I/2010 (IBSP 175440, IBSP 175592, IBSP 175593) 1♀, 01–14/VII/2010 (IBSP 175591), all collected by R. Andrade & I. Cizauskas et al.

#### Etymology.

The specific name refers to Atlach-Nacha, a supernatural entity from Cthulhu mythology that resembles a huge spider with an almost human face.

#### Diagnosis.

*Ochyrocera atlachnacha* resembles *O. varys* by its carapace yellow and bright lime (Figs 1A–B, 4A–B) and palp with conical, elongated and distal cuspule in the cymbial apophysis (Figs 1C–D, 4C–D). It can be distinguished from the latter and other Neotropical species by the male palpal cymbium with accentuated cymbial prolateral projection (Figs 4D, 5C, E–F); females have enlarged and projected pore-plates on the inconspicuous spermathecae (Fig. 4E–F).

#### Description.

**Male** (holotype). Total length 2.2 Carapace length 0.7, ovoid; narrowing gradually anteriorly; yellow and bright lime, flat pars cephalic, fovea not visible (Fig. 4A). Clypeus length 0.7, with a pair of long bristles (Fig. 4A). Eyes: PME oval; ALE and PLE rounded. Chelicerae light yellow; promargin with 6 teeth, attached to a very long lamina (Fig. 5A); retromargin without teeth. Sternum light gray to darker. Endites dark yellow to green. Legs: gray; formula 1423; total length: I 7.0; II 5.9; III 4.1; IV 6.5. Male palp: palpal femur length 0.4; palpal tibia enlarged basally; cymbium slightly curved distally, bearing short apical cuspule; retrolateral paired long setae on non-projected base and an elongated tarsal organ (Fig. 5D) with three basal setae on the rounded cymbial prolateral extension (Fig. 5C); bulb oval; embolus flattened, short, with sinuous tip (Figs 4C–D, 5C–F). Abdomen length 1.3, oval; uniformly grayish-green. Six epiandrous spigots, with short base (Fig. 5B)

**Female** (paratype IBSP 188899). Total length: 2.0; carapace length: 0.74; Carapace as in male, light yellow pattern (Fig. 4B). Pedipalp without claw, with conical tip and subdistal trichobothrium (Fig. 6A–B). Clypeus: 0.68 diameter. Eyes, chelicerae, sternum, endites (Fig. 6C) and labium as in male. Legs as in male; formula 4123, total length: I 6.3; II 4.7; III 3.6 IV4.3. Abdomen length 0.96. Colulus triangular with approximately 10 bristles (Fig. 6D). Internal genitalia with inconspicuous and very narrow spermathecae, under the conspicuous pore-plate; short medial columnar uterus externus, internally with no visible chambers. Uterus externus ending in a narrow neck. Oval pore-plates on the spermathecae, with approximately 25–30 glandular ducts (Fig. 4E–F).

#### Distribution.

Recorded exclusively from caves in Carajás, state of Pará, northern Brazil (Fig. 20B).

### Ochyrocera laracna sp. n.

Taxon classificationAnimaliaAraneaeOchyroceratidae

http://zoobank.org/9DA06567-8A56-4C0D-A84E-864BC55E38A5

**Figure 7.**
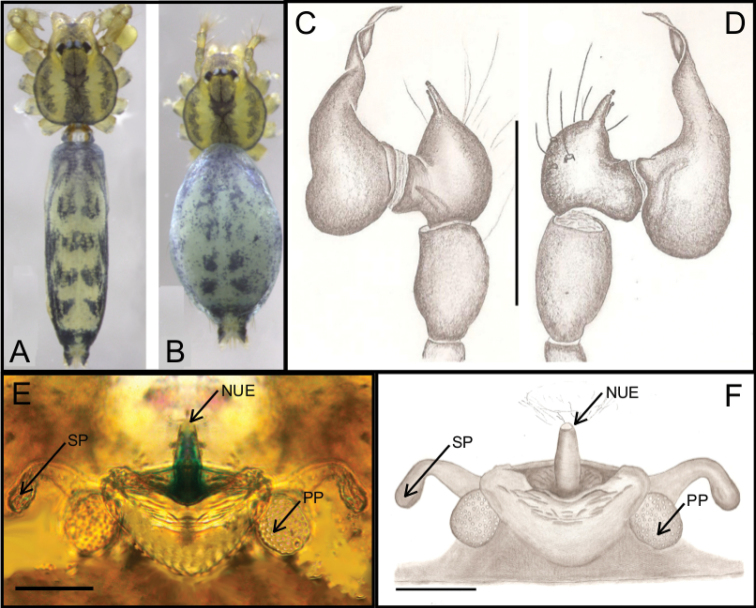
*Ochyrocera laracna* sp. n. male holotype (**A, C–D**), female paratype, IBSP 191335 (**B, E–F**) **A–B** habitus, dorsal view **C** left male palp, retrolateral view **D** same, prolateral view **E** genitalia, enzyme cleared, dorsal view **F** same, dorsal view. Abbreviations: NUE = neck of uterus externus, PP = pore-plate, SP = spermathecae. Scale bars: 0.5 mm (**A, B**); 0.7 mm (**C, D**); 500 µm (**E, F).**

**Figure 8.**
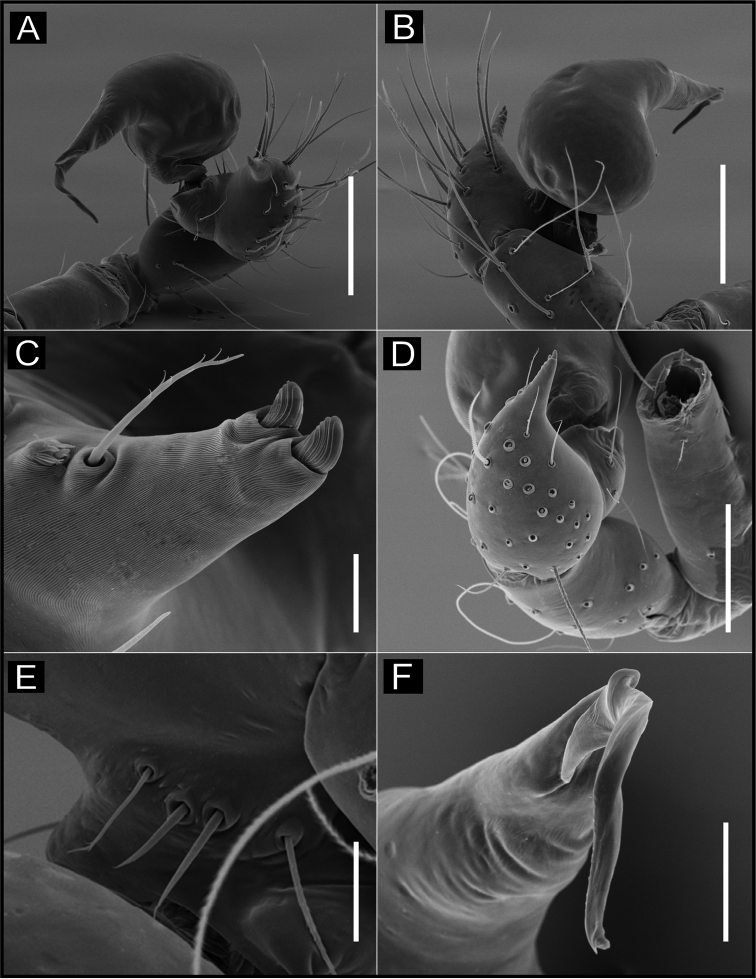
SEM images of *Ochyrocera laracna* sp. n., male IBSP 191335 (**A–F**) **A** left palp, retroapical view **B** same, prolateroapical view **C** cymbium, distal view, double cuspules, long hair and tarsal organ **D** cymbium, dorsal view **E** same, basal setae **F** embolus, distal area. Scale bars: 0.3 mm.

**Figure 9.**
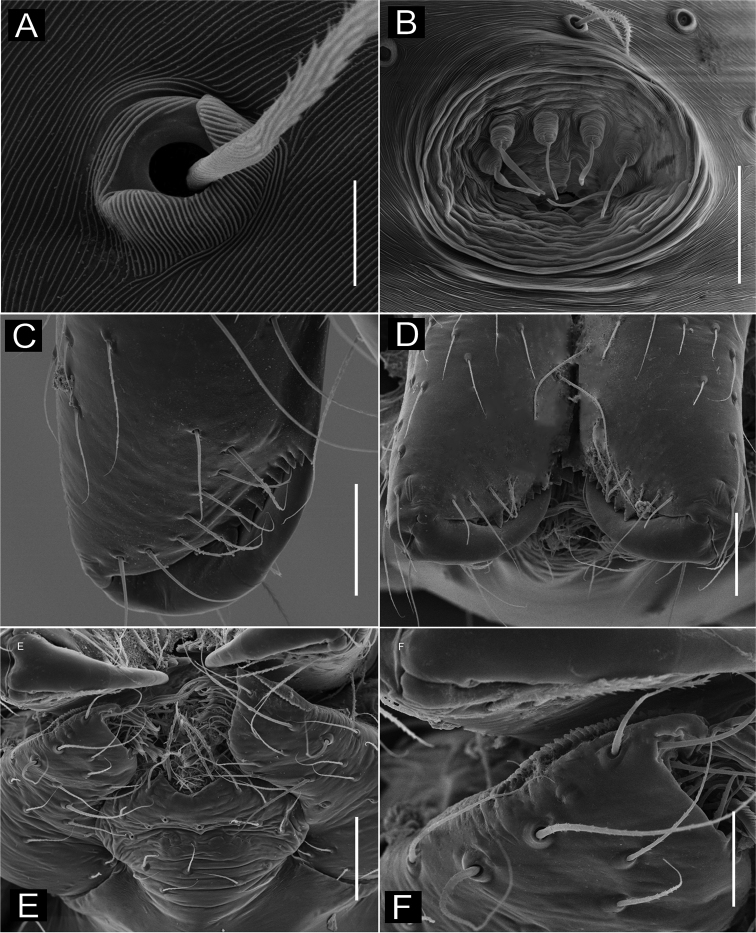
SEM images of *Ochyrocera laracna* sp. n., male IBSP 191335 (**A–C**), female IBSP 191335 (**D–F**) **A** trichobothria, palp, dorsal view **B** epiandrous area, abdomen, ventral view **C** chelicerae, frontal view **D** same, frontal view **E** endites and labium, ventral view **F** serrula, ventral view. Scale bars: 300 µm.

Figures 7 , 8 , 9 , 20A

#### Types.

Holotype male from Cave N5S-15/16 (50°7'60"W, 6°6'20"S), Serra Norte, Floresta Nacional de Carajás, Parauapebas, Pará, Brazil, 14–23/X/2009, R. Andrade & I. Cizauskas et al. (IBSP 177631). Paratypes: Cave N4E_13 (6°2'18"S; 50°9'38"W), Serra Norte, Floresta Nacional de Carajás, Parauapebas, Pará, Brazil, 20/X−01/XI/2006, R. Andrade (IBSP 188898).

#### Other examined material.

BRAZIL. Pará: **HYPOGEAN SAMPLES**: Canaã dos Carajás, Floresta Nacional de Carajás, Serra Sul, Cave S11D-64 (6°23'31"S; 50°18'48"W), 2♂4♀, 13–30/I/2010 (IBSP 174071); Cave CAV_0014 (6°24'59"S; 50°19'15"W), 1♀, 22–28/IX/2010 (IBSP 174075); Cave CAV-19 (6°29'52"S; 51°9'55"W), 1♀, 08–15/III/2012 (IBSP 175981); 1♂, 20–29/VI/2012 (IBSP 175999); Cave CAV_06 (6°29'51"S; 51°9'44"W), 1♀, 08–15/III/2012 (IBSP 176047); Cave CAV_18 (6°29'50"S; 51°9'31"W), 1♀, 08–15/III/2012 (IBSP 176065); Cave CAV_03 (6°29'39"S; 51°9'48"W), 1♀, 20–29/VI/2012 (IBSP 176071); Cave CAV_06 (6°29'51"S; 51°9'44"W), 1♀, 20–29/VI/2012 (IBSP 176073), all collected by R. Andrade & I. Cizauskas et al.; Parauapebas, Cave CRIS_38 (6°27'32"S; 49°42'13"W), 1♀, 29/VII-06/VIII/2008 (IBSP 174645); collected by R. Andrade et al.; Floresta Nacional de Carajás, Serra Norte, Cave N5S_no number (6°5'16"S; 50°7'11"W), 1♀, 28/IX-03/X/2007, collected by R. Andrade & I. Arnori et al. (IBSP 191356); Cave N5S_15/16 (6°6'20"S; 50°7'60"W), 1♂, 14–23/X/2009 (IBSP 177631), collected by R. Andrade & I. Cizauskas et al.; Cave N3_0070 (6°2'39"S; 50°13'48"W), 1♀, 03–17/IV/2013 (IBSP 178566); Cave N3_0078 (6°2'36"S; 50°13'43"W), 1♂, 03–17/IV/2013 (IBSP 178567), all collected by Equipe Carste et al.; Cave N5SM1_0004 (-6,112018; -50,135539), 1♂, 04/IX−06/X/2014 (ISLA 14615); Cave N5SM2_0058 (6°7'46"S; 50°8'5"W), 1♂, 24/II−13/III/2015 (ISLA 14617), all collected by Bioespeleo et al.; **EPIGEAN SAMPLES**: Parauapebas, Floresta Nacional de Carajás, Serra Norte (6°5'15"S; 50°7'12"W), 1♀, 25/I/2012 (IBSP 191368); (6°5'16"S; 50°7'36"W), 2♀, 19/XI/2012 (IBSP 191352, IBSP 191356); (6°5'18"S; 50°7'11"W), 5♂, 25/IV-03/V/2012 (IBSP 191366, IBSP 191367, IBSP 191380, IBSP 191385), 2♂3♀, 08–14/XI/2012 (IBSP 191354, IBSP 191353, IBSP 191365); (6°5'18"S; 50°7'12"W) 1♂, 25/I/2012 (IBSP 191371); (6°5'20"S; 50°7'10"W), 2♂1♀, 08–14/XI/2012 (IBSP 191369, IBSP 191384); (6°5'20"S; 50°7'10"W), 12♂, 25/IV-03/V/2012 (IBSP 191360, IBSP 191362, IBSP 191383, IBSP 191387, IBSP 191391, IBSP 191392, IBSP 191364, IBSP 191372, IBSP 191376); (6°6'12"S; 50°7'45"W), 1♂, 26/IV-03/V/2012 (IBSP 191382) 1♀, 27/I/2012 (IBSP 191355); (6°6'17"S; 50°7'48"W), 1♂, 26/IV−-03/V/2012 (IBSP 191388); (6°6'20"S; 50°7'41"W), 1♂1♀, 16–20/XI/2012 (IBSP 191361, IBSP 191378); (6°6'20"S; 50°7'55"W), 1♀, 27/I/2012 (IBSP 191370); (6°6'22"S; 50°7'47"W), 9♂1♀, 25/IV-03/V/2012 (IBSP 191379, IBSP 191363, IBSP 191377, IBSP 191389, IBSP 191390, IBSP 191373, IBSP 191358) 2♂1♀, 16–20/XI/2012 (IBSP 191386, IBSP 191374, IBSP 191375); (6°6'24"S; 50°7'55"W), 1♀, 25/IV-03/V/2012 (IBSP 191359); all collected by I. Cizauskas & R. Andrade et al.; (6°0'59"S; 50°4'43"W), 1♂2♀, 9/III/2013 (IBSP 191396, IBSP 191402); (6°1'46"S; 50°12'4"W), 2♀, 13/X/2012 (IBSP 191400, IBSP 191401); (6°2'33"S; 50°13'23"W), 2♂, 11/III/2013 (IBSP 191397); (6°2'33"S; 50°13'6"W), 1♀, 5/III/2013 (IBSP 191399); (6°3'24"S; 50°4'49"W), 1♂, 6/III/2013 (IBSP 191403); (6°3'9"S; 50°14'31"W); 1♂, 14/III/2013 (IBSP 191398); 1♂, 12/X/2012 (IBSP 191332); all collected by Equipe Carste et al.

#### Etymology.

The specific name refers to Laracna, a giant and very old spider created by J. R. R. Tolkien in the classic book “The Lord of the Rings”.

#### Diagnosis.

*Ochyrocera laracna* resembles *O. aragogue* by the yellowish-green body pattern (Figs 7A–B; 8A−B) and by the short cymbial apophysis with two distal spurs on projections (Fig. 7C–D), a unique character for both these Neotropical species. The male of the former species can be distinguished from the latter due to the palp having a flap at the distal area of embolus (Figs 7C–D; 8A−B) and a laminar spur in the curved area 8F). The female is distinguished from *O. aragogue* by the small distal area of the spermathecae and pore plates adjacent to the spermathecae base (Fig. 7E–F).

#### Description.

**Male** (holotype). Total length 2.2. Carapace length 0.7, ovoid; narrowing gradually anteriorly, with yellowish-green body pattern, pars cephalica flat, fovea not visible (Fig. 7A). Clypeus length 0.7, curved forward. Eyes: PME oval; ALE and PLE rounded. Chelicerae light yellow, promargin with seven teeth, attached to long lamina (Fig. 9C); retromargin without teeth. Sternum light yellow to slightly darker. Endites dark yellow suffused. Legs light yellow; formula 1423; total length: I 7.0; II 5.9; III 4.1; IV 6.5. Male palp: palpal femur length 0.4; palpal tibia enlarged medially with dorsal trichobothrium (Fig. 9A); cymbial apophysis short, bearing parallel double short cuspules at tip, retrolateral long hair next to the tarsal organ (Fig. 8C); with four basal setae (Fig. 8E) and cymbial prolateral extension squared (8A); bulb oval; embolus enlarged at base (Figs 7C–D, 8A–F). Abdomen length 1.3, oval; uniformly green-purplish with black spots. Six epiandrous spigots, with short base (Fig. 9B).

**Female** (paratype IBSP 188898). Total length 2.0; carapace length: 0.74; Carapace as in male (Fig. 7B). Pedipalp without claw, with conical tip and subdistal trichobothria. Clypeus: 0.68 diameter. Eyes, chelicerae, sternum, endites and labium as in male, serrula with more than 50 teeth (Fig. 9E–F). Legs as in male; leg formula 4123, total length: I 6.3; II 4.7; III 3.6 IV 4.3. Abdomen length 0.96. Colulus triangular, with 8–10 bristles. Internal genitalia weakly sclerotized, spermathecae tubular, thicker at basal area and curved at middle; elongated medial columnar uterus externus, shorter than spermathecae, internally with inconspicuous chambers. Uterus externus ending in a narrow neck. Oval pore-plates at the spermathecae base, with approximately 40–50 glandular ducts (Figs 7E–F).

#### Distribution.

Recorded from caves and epigean areas in the Carajás region, state of Pará, northern Brazil (Fig. 20A).

### Ochyrocera aragogue sp. n.

Taxon classificationAnimaliaAraneaeOchyroceratidae

http://zoobank.org/E9FA58F0-3F9B-4468-AB05-BD26723FCBDB

**Figure 10.**
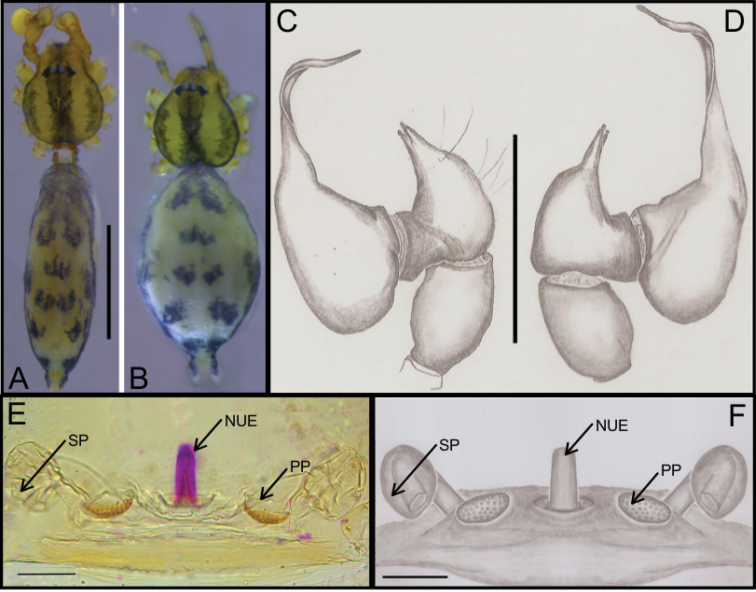
*Ochyrocera aragogue* sp. n., male holotype (**A, C, D**), female paratype, IBSP 188850 (**B, E, F**) **A, B** habitus, dorsal view **C** left male palp, retrolateral view **D** same, prolateral view **E** genitalia, enzyme cleared, dorsal view **F** same, dorsal view. Abbreviations: NUE = neck of uterus externus, PP = pore-plate, SP = spermathecae. Scale bars: 0.5 mm (**A, B**); 0.7 mm (**C, D**); 500 µm (**E, F**).

**Figure 11.**
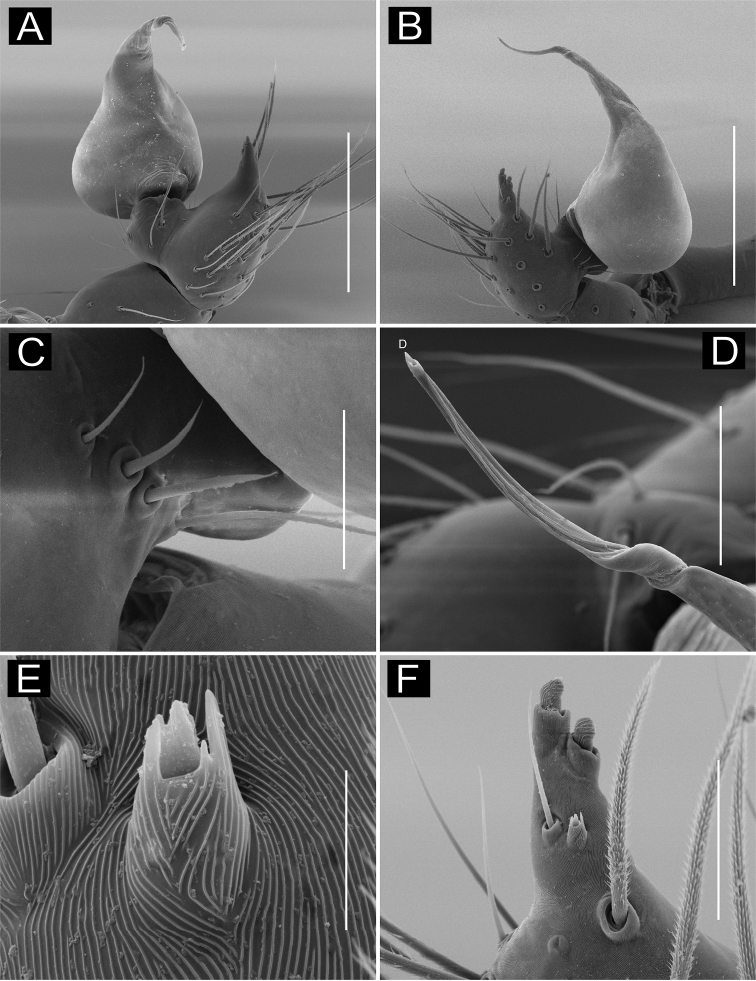
SEM images of *Ochyrocera aragogue* sp. n., male IBSP 188851 (**A–F**) **A** left palp, retrolateral view **B** same, prolateral view **C** cymbium, basal setae **D** embolus, distal area **E** cymbium, tarsal organ **F** same, cuspules, long hair and tarsal organ, distal tip. Scale bars: 0.3 mm (**A–D**); 5 µm (**E**); 30 µm (**F**).

**Figure 12.**
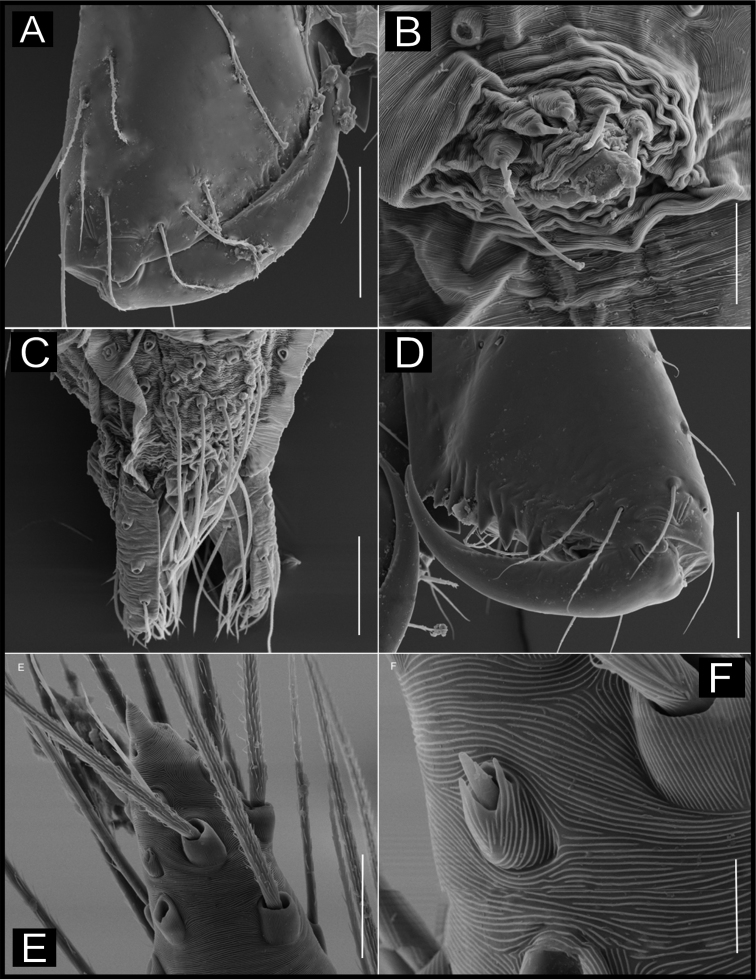
SEM images of *Ochyrocera aragogue* sp. n., male IBSP 188851 (**A–B**), female, IBSP 188850 (**C–F**) **A** chelicerae, frontal view, **B** epiandrous area, abdomen, ventral view **C** colulus, ventral view **D** chelicerae, frontal view **E** pedipalp, distal, prolateral view **F** same, tarsal organ. Scale bars: 0.3 mm (**A–D**); 20 µm (**E**); 5 µm (**F**).

Figures 10 , 11 , 12 , 19B

#### Types.

Holotype male from Cave N4E_0008 (50°9'36"W, 6°2'21"S), Serra Norte, Floresta Nacional de Carajás, Parauapebas, Pará, Brazil, 07–12/X/2008, R. Andrade et al. (IBSP 174962). Paratype female from Cave N4E_0013 (6°2'18"S; 50°9'38"W), Serra Norte, Floresta Nacional de Carajás, Parauapebas, Brazil, 20/X_01/XI/2006, R. Andrade & I. Arnori et al. (IBSP 174983).

#### Other examined material.

BRAZIL. Pará: **HYPOGEAN SAMPLES**: Curionópolis, Serra Leste, Cave SL_no number (5°58'35"S; 49°37'55"W), 1♀, 17–24/X/2008, R. Andrade et al. (IBSP 188853); Parauapebas, Floresta Nacional de Carajás, Serra Norte, Cave N4E_0008 (6°2'21"S; 50°9'36"W), 1♂, 07–12/X/2008, R. Andrade et al. (IBSP 174962); Cave N3_0070 (6°2'39"S; 50°13'48"W), 1♀, 03–17/IV/2013 (IBSP 174076); Cave N3_0078 (6°2'36"S; 50°13'43"W), 1♂, 03–17/IV/2013 (IBSP 174077); Cave N1_0125 (6°0'15"S; 50°17'15"W), 1♀, 07–28/I/2015 (IBSP 188850); Cave N1_0170 (6°1'23"S; 50°17'58"W), 1♂, 03–17/XII/2014 (IBSP 188851); Cave N1_0038 (6°1'49"S; 50°16'17"W), 1♀, 04/IX–06/X/2014 (IBSP 188852), all collected by Equipe Carste et al.

#### Etymology.

The specific name refers to Aragog, a spider capable of communicating with humans and a lover of human flesh, from the literary classic “Harry Potter and the Chamber of Secrets”, by J.K. Rowling.

#### Diagnosis.

*Ochyrocera aragogue* resembles *O. laracna* by the yellowish green body color pattern (Fig. 10A–B) and by the short cymbial apophysis with two distal cuspules on projections (Fig. 10C–D, 11F), a unique character for both these Neotropical species. The male can be distinguished from *O. laracna* by the palp with a sinuous distal area of embolus without laminar spur (Figs 10C–D, 11A–B, D). The female has an enlarged distal area of spermathecae and pore plates at the spermathecae base (Fig. 10E–F).

#### Description.

**Male** (holotype). Total length 2.3. Carapace length 0.7, ovoid; narrowing gradually anteriorly, with yellowish-green pattern, pars cephalica flat, fovea not visible (Fig. 10A). Clypeus length 0.7, curved foward. Eyes: PME elongated oval; ALE and PLE rounded. Chelicerae light yellow, promargin with seven teeth, attached to long lamina (Fig. 12A); retromargin without teeth. Sternum light yellow gray. Endites dark yellow suffused. Legs light yellow, formula 1423, total length I 7.0; II 5.9; III 4.1; IV 6.5. Male palp: palpal femur length 0.4; palpal tibia short, as long as cymbium; cymbial apophysis short, bearing two short distal cuspules at tip, in different heights, retrolateral long hair on non-projected base, next to the tarsal organ; with four basal setae (Fig. 11C, E–F); cymbial prolateral extension almost squared; embolus elongated, enlarged at base and subapically twisted (Figs 10C–D, 11A). Abdomen length 1.3, oval, uniformly green-purplish color (Fig. 9A). Six epiandrous spigots, with short base (Fig. 12B).

**Female** (paratype IBSP 174983). Total length 2.0; carapace length 0.74, pattern light yellowish (Fig. 10B). Pedipalp without claw, with conical tip and subdistal tarsal organ (Fig. 12E–F). Clypeus 0.68 diameter. Eyes, chelicerae, sternum, endites (Fig. 12D) and labium as in male. Legs as in male, formula 4123, total length I 6.3; II 4.7; III 3.6 IV 4.3. Abdomen length 0.96, globular (Fig. 10B). Colulus triangular, with approximately 10 bristles (Fig. 12C). Internal genitalia weakly sclerotized, spermathecae tubular, slender in basal area and curved and thickened at distal area. Uterus externus shorter than spermathecae, internally with no visible chambers, ending in truncated neck. Oval pore-plates at the base of spermathecae, with approximately 25–30 glandular ducts (Fig. 10E–F).

#### Distribution.

Recorded exclusively from caves in the Carajás region, state of Pará, northern Brazil (Fig. 19B).

### Ochyrocera misspider sp. n.

Taxon classificationAnimaliaAraneaeOchyroceratidae

http://zoobank.org/A4A64C11-B4B6-4B3B-9E95-323239941030

**Figure 13.**
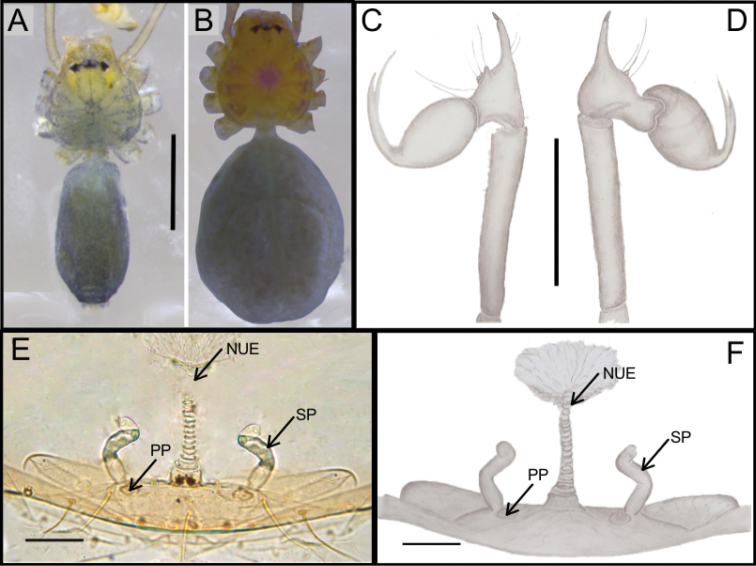
**A–F** *Ochyrocera misspider* sp. n., male holotype (**A, C, D**), female paratype, IBSP 176870 (**B, E, F**) **A, B** habitus, dorsal view **C** left male palp, retrolateral view **D** same, prolateral view **E** genitalia, enzyme cleared, dorsal view **F** same, dorsal view. Abbreviations: NUE = neck of uterus externus, PP = pore-plate, SP = spermathecae. Scale bars: 0.5 mm (**A, B**); 0.7 mm (**C, D**); 500 µm (**E, F**).

**Figure 14.**
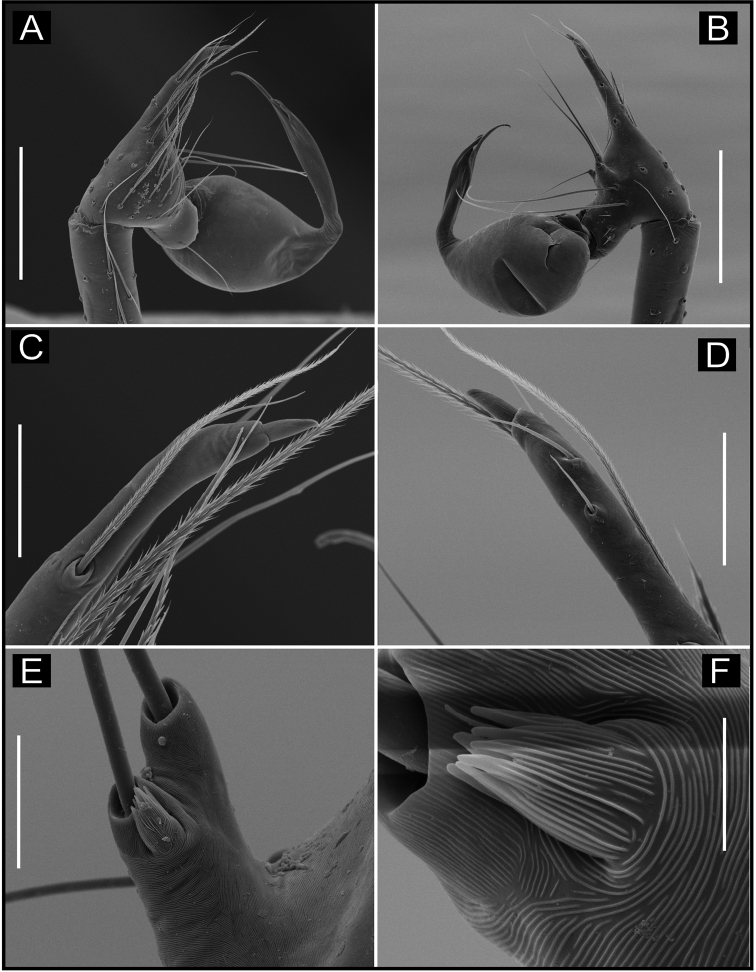
SEM images of *Ochyrocera misspider* sp. n., male IBSP176911 (**A–F**) **A** palp, prolateral view **B** same, retrolateral view **C** same, cymbium, distal area, prolateral view **D** same, cymbium, retrolateral view **E** cymbium, spur with long hairs and tarsal organ **F** same, tarsal organ, detail. Scale bars: 0.3 mm (**A–D**); 20 µm (**E**); 5 µm (**F**).

**Figure 15.**
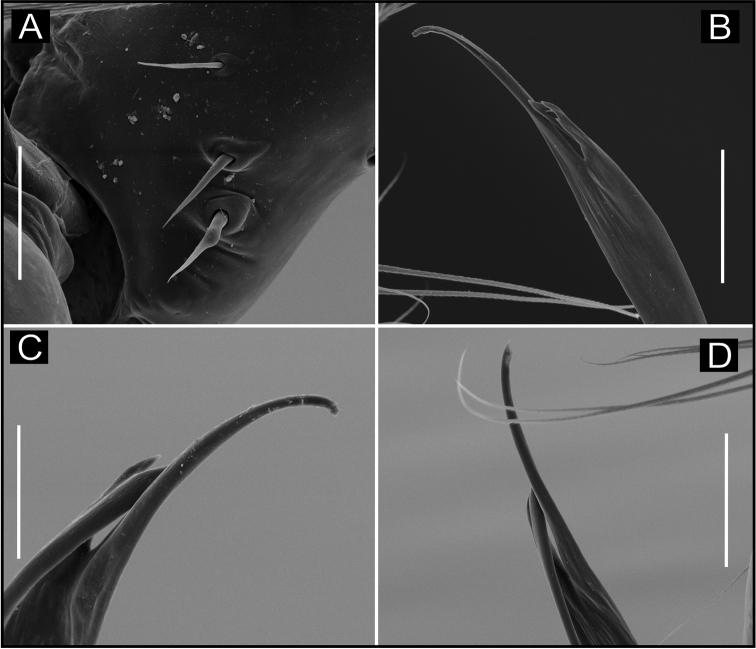
SEM images of *Ochyrocera misspider* sp. n., male palp, IBSP 176911(**A–D**) **A** cymbium, basal setae (**B–D**) embolus, distal tip **B** prolateral view **C** retrolateral view **D** ventral view Scale bars: 0.5 mm (**A, B**); 0.7 mm (**C, D**); 500 µm (**E, F**).

**Figure 16.**
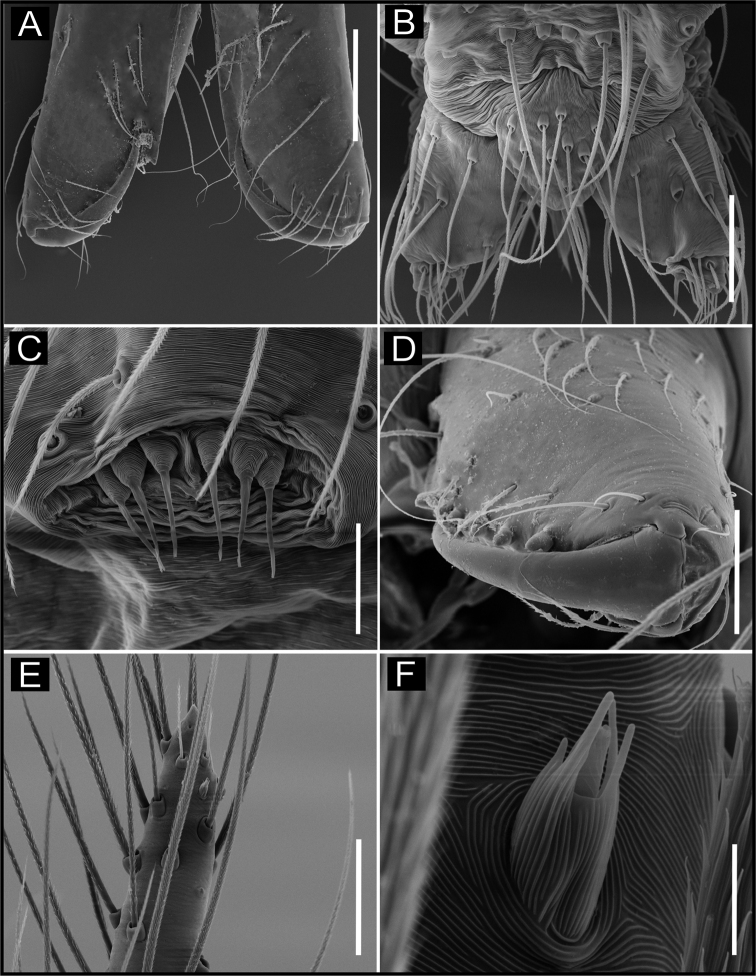
SEM images of *Ochyrocera misspider* sp. n. male IBSP 176911 (**A, C**), female, IBSP 178561 (**B, D–F**) **A** chelicerae, frontal view, **B** colulus, ventral view **C** epiandrous, abdomen, ventral view **D** chelicerae, frontal view **E** pedipalp, distal, prolateral view **F** same, tarsal organ. Scale bars: 0.5 mm (**A, B**); 0.7 mm (**C, D**); 500 µm (**E, F**).

Figures 13 , 14 , 15 , 16 , 19B , 21C

#### Types.

Holotype male from Cave N4E_0070 (6°1'56"S, 50°9'10"W), Serra Norte, Floresta Nacional de Carajás, Parauapebas, Pará, Brazil, 24–30/VII/2009, R. Andrade & I. Cizauskas et al. (IBSP 176910). Paratype: female from Cave N4E_0070 (6°1'56"S, 50°9'10"W), Serra Norte, Floresta Nacional de Carajás, Parauapebas, Pará, Brazil, 19/II-04/III/2010, R. Andrade & I. Cizauskas et al. (IBSP 176870).

#### Other material examined.

BRAZIL. Pará: **HYPOGEAN SAMPLES**: Canaã dos Carajás, Floresta Nacional de Carajás, Serra Sul, Cave CAV_0024 (6°24'20"S; 50°21'57"W), 1♀, 22–31/V/2010 (IBSP 175310), 2♀, 22–28/IX/2010 (IBSP 175314); Cave CAV_0032 (6°25'35"S; 50°19'25"W), 1♀, 22–28/IX/2010 (IBSP 175315); Cave S11D-101 (6°23'22"S; 50°21'48"W), 1♀, 01–14/VII/2010 (IBSP 175311); Cave S11D-26 (6°24'49"S; 50°21'17"W), 1♀, 19–22/II/2010 (IBSP 175309); Cave S11D-31 (6°24'41"S; 50°20'43"W), 3♀, 03–19/VIII/2010 (IBSP 175312); Cave S11D-39 (6°23'46"S; 50°20'27"W), 1♀, 03–19/VIII/2010 (IBSP 175313); 5♀, 13–30/I/2010 (IBSP 175307, IBSP 175306, IBSP 175308); Cave S11D-89 (6°23'45"S; 50°19'20"W), 3♀, 13–30/I/2010 (IBSP 175305); Cave S11D_94 (6°23'40"S; 50°19'17"W), 1♀, 13–30/I/2010 (IBSP 175304), all collected by R. Andrade & I. Cizauskas et al.; Parauapebas, Cave CRIS_20 (6°25'35"S; 49°41'18"W), 6♀, 29/VII-06/VIII/2008, R. Andrade (IBSP 174597); Floresta Nacional de Carajás, Serra Norte, Cave GEM_1570, 3♀, 17–24/X/2008, R. Andrade (IBSP 174516); Cave N1_0025 (6°1'54"S; 50°16'21"W), 1♀, 02–29/IV/2015 (IBSP 188874); Cave N1_0039 (6°1'46"S; 50°16'13"W), 1♀, 02–29/IV/2015 (IBSP 188873); Cave N1_0169 (6°1'23"S; 50°17'59"W), 1♀, 03–17/XII/2014 (IBSP 188870); Cave N1_0176 (6°1'29"S; 50°18'2"W), 1♀, 02–23/IV/2015 (IBSP 188872); Cave N1_0180 (6°2'33"S; 50°16'25"W), 2♀, 28/IX-03/X/2007 (IBSP 174731); Cave N1_0221 (6°1'48"S; 50°18'2"W), 1♀, 04/IX-06/X/2014 (IBSP 188869); Cave N1-0106 (6°0'46"S; 50°18'22"W), 1♀, 07–28/I/2015 (IBSP 188871); Cave N1-0226 (6°2'16"S; 50°16'2"W), 1♀, 02–29/IV/2015 (IBSP 188875); Cave N2_026 (6°3'16"S; 50°14'23"W), 3♀, 26/IX-17/X/2012 (IBSP 178500, IBSP 178498); Cave N3_0002 (6°1'43"S; 50°12'2"W), 1♀, 05–17/III/2013 (IBSP 178503); Cave N3_0003 (6°1'44"S; 50°12'3"W), 1♀, 26/IX-17/X/2012 (IBSP 178480); Cave N3_0004 (6°1'45"S; 50°12'2"W), 1♀, 26/IX-17/X/2012 (IBSP 178483); Cave N3_0006 (6°1'45"S; 50°12'3"W), 1♀, 26/IX-17/X/2012 (IBSP 178484); Cave N3_0023 (6°2'35"S; 50°13'10"W), 1♀, 02–23/VIII/2013 (IBSP 178538); Cave N3_0026 (6°2'39"S; 50°13'9"W), 4♀, 26/IX-17/X/2012 (IBSP 178487, IBSP 178488, IBSP 178490), 1♀, 05–17/III/2013 (IBSP 178511); Cave N3_0028 (6°2'32"S; 50°13'5"W), 1♀, 05–17/III/2013 (IBSP 178513); Cave N3_0033 (6°2'42"S; 50°13'12"W), 1♀, 26/IX-17/X/2012 (IBSP 178493); Cave N3_0037 (6°2'45"S; 50°13'14"W), 1♀, 26/IX-17/X/2012 (IBSP 178496); Cave N3_0047 (6°2'27"S; 50°13'40"W), 5♀, 03–17/IV/2013 (IBSP 178526, IBSP 178528, IBSP 178530); 1♀, 02–23/VIII/2013 (IBSP 178542); Cave N3_0054 (6°2'25"S; 50°13'42"W), 3♀, 02–23/VIII/2013 (IBSP 178543, IBSP 178544, IBSP 178545); Cave N3_0074 (6°2'35"S; 50°13'49"W), 4♀, 05–17/III/2013 (IBSP 178520, IBSP 178522, IBSP 178523); 2♀, 02–23/VIII/2013 (IBSP 178547, IBSP 178550); Cave N3_0076 (6°2'28"S; 50°13'36"W), 1♂1♀, 02–23/VIII/2013 (IBSP 178551); 2♀, 03–17/IV/2013 (IBSP 178535, IBSP 178536); Cave N5SM2_0081 (6°7'19"S; 50°7'44"W) 2♀, 2010–11 (ISLA 14622); Cave N5W-03 (64'53"S; 50°8'4"W) 2♀, 02–23/VIII/2013 (IBSP 178556, IBSP 178561); Cave N8_0002 (6°10'5"S; 50°9'35"W), 4♀, 02–29/IV/2015 (IBSP 188876); Cave N8_0003 (6°10'6"S; 50°9'32"W), 2♀, 02–29/IV/2015 (IBSP 188877); Cave N8_0018 (6°10'8"S; 50°9'28"W), 1♀, 02–29/IV/2015 (IBSP 188878); Cave N8_0019 (6°10'11"S; 50°9'27"W), 1♀, 02–29/IV/2015 (IBSP 188879); Cave N8_0022 (6°10'6"S; 50°9'30"W), 1♀, 02–29/IV/2015 (IBSP 188895); Cave N8–0025 (6°10'29"S; 50°9'4"W), 1♀, 02–29/IV/2015 (IBSP 188880); all collected by Equipe Carste et al.; Cave N4E_0002 (6°2'25"S; 50°9'39"W), 1♀, 20/IV-04/V/2010 (IBSP 176940); Cave N4E_0003 (6°2'25"S; 50°9'38"W), 2♀, 20/IV-04/V/2010 (IBSP 176941, IBSP 176942); Cave N4E_0007 (6°2'21"S; 50°9'36"W), 2♀, 20/IV-04/V/2010 (IBSP 176943, IBSP 176944); Cave N4E_0008 (6°2'21"S; 50°9'36"W), 10♀, 20/IV-04/V/2010 (IBSP 176948, IBSP 176949, IBSP 176950, IBSP 176951, IBSP 176946, IBSP 176947); Cave N4E_0010 (6°2'20"S; 50°9'38"W), 1♀, 20/IV-04/V/2010 (IBSP 176952); Cave N4E_0011 (6°2'20"S; 50°9'38"W), 3♀, 20/IV-04/V/2010 (IBSP 176953); Cave N4E_0015 (6°2'10"S; 50°9'35"W), 1♀, 20/IV-04/V/2010 (IBSP 176945); Cave N4E_0022 (6°2'2"S; 50°10'4"W), 1♀, 20/IV-04/V/2010 (IBSP 176954); Cave N4E_0033 (6°2'25"S; 50°9'36"W) 1♀, 15–22/IX/2009 (IBSP 176927); Cave N4E_0043 (6°1'55"S; 50°9'50"W) 1♀, 19/II-04/III/2010 (IBSP 176862); Cave N4E_0045 (6°2'25"S; 50°9'40"W), 1♀, 24–30/VII/2009 (IBSP 176928); Cave N4E_0047 (6°2'15"S; 50°9'36"W), 1♂, 18/VIII-03/IX/2009 (IBSP 176912); Cave N4E_0048 (6°2'15"S; 50°9'37"W), 1♀, 19/II-04/III/2010 (IBSP 176931); Cave N4E_0051 (6°2'22"S; 50°9'38"W), 1♀, 19/II-04/III/2010 (IBSP 176932); Cave N4E_0053 (6°2'3"S; 50°10'2"W), 1♀, 24–30/VII/2009 (IBSP 176929); Cave N4E_0055 (6°1'55"S; 50°9'59"W), 2♀, 19/II-04/III/2010 (IBSP 176930); Cave N4E_0070 (6°1'56"S; 50°9'10"W), 1♀, 19/II-04/III/2010 (IBSP 176870); Cave N4E_0070 (6°1'56"S; 50°9'10"W); 1♂1♀, 24–30/VII/2009 (IBSP 176918, IBSP 176910); Cave N4E_0072 (6°1'56"S; 50°9'13"W), 1♂2♀, 24–30/VII/2009 (IBSP 176911, IBSP 176921); 1♂8♀, 19/II-04/III/2010 (IBSP 176871, IBSP 176873, IBSP 174074); Cave N4E_0074 (6°1'59"S; 50°9'21"W), 2♀, 19/II-04/III/2010 (IBSP 176933, IBSP 176934); Cave N4E_0079 (6°1'59"S; 50°9'5"W), 5♀, 19/II-04/III/2010 (IBSP 176936, IBSP 176935); Cave N4E_0080 (6°1'58"S; 50°9'4"W), 3♀, 24–30/VII/2009 (IBSP 176920, IBSP 176919); 6♀, 19/II-04/III/2010 (IBSP 174064); Cave N4E_0085 (6°2'3"S; 50°9'26"W), 1♀, 19/II-04/III/2010 (IBSP 176937); Cave N4E_0092 (6°2'22"S; 50°9'31"W), 6♀, 24–30/VII/2009 (IBSP 176924, IBSP 176926, IBSP 176923, IBSP 176925); 2♀, 19/II-04/III/2010 (IBSP 176938, IBSP 176939); Cave N4E_0093 (6°2'22"S; 50°9'31"W), 2♀, 24–30/VII/2009 (IBSP 176922); Cave N5S_04 (6°6'20"S; 50°8'2"W), 1♀, 14–23/X/2009 (IBSP 177621); Cave N5S_07 (6°6'20"S; 50°7'59"W), 7♀, 14–23/X/2009 (IBSP 177616, IBSP 177617, IBSP 177619, IBSP 177618, IBSP 177620); Cave N5S_12 (6°6'11"S; 50°7'31"W) 4♀, 14–23/X/2009 (IBSP 177622, IBSP 177623); Cave N5S_37 (6°6'22"S; 50°7'57"W), 1♀, 14/III-04/IV/2010 (IBSP 177624); Cave N5S_40 (6°6'19"S; 50°8'0"W), 2♀, 15–21/IX/2009 (IBSP 177615); Cave N5S_52/53 (6°6'28"S; 50°7'59"W), 2♀, 25/VIII-03/IX/2009 (IBSP 177613, IBSP 177614); Cave N5S_55 (6°6'28"S; 50°7'57"W), 1♀, 25/VIII-03/IX/2009 (IBSP 177610); 1♀, 14/III-04/IV/2010 (IBSP 177625); Cave N5S_63/64/65 (6°6'12"S; 50°8'7"W), 1♀, 14/III-04/IV/2010 (IBSP 177626); Cave N5S_68 (6°6'3"S; 50°8'7"W), 1♀, 25/VIII-03/IX/2009 (IBSP 177608); Cave N5S_70 (6°6'5"S; 50°8'4"W), 3♀, 25/VIII-03/IX/2009 (IBSP 177611, IBSP 177612, IBSP 177670) 2♀, 14/III-04/IV/2010 (IBSP 177627); Cave N5S_74 (6°6'2"S; 50°8'5"W), 1♀, 14/III-04/IV/2010 (IBSP 177628); Cave N5S-79 (6°6'8"S; 50°8'13"W), 2♀, 14/III-04/IV/2010 (IBSP 177629); Cave N5S_85 (6°5'12"S; 50°7'35"W), 1♀, 25/VIII-03/IX/2009 (IBSP 177609); all collected by R. Andrade & I. Cizauskas et al.

#### Etymology.

The specific name refers to Little Miss Spider, a very popular spider around the world and the main character of the children’s books by David Kirk.

#### Diagnosis.

*Ochyrocera misspider* is the smallest among the species from Floresta Nacional de Carajás and resembles *O. caeruleoamethystina* Lopez & Lopez and *O. thibaudi* Emerit & Lopez by the small projection in the cymbium (see Lopez and Lopez 1997, fig. 8; Emerit and Lopez 1985, fig. 1A). It can be distinguished by the male palp with an elongated tibia, twice as long as the cymbium, and by the bifid embolus (Figs 13C–D, 14A–B). Females are distinguished from other species of the genus by the genitalia with a very long and narrow medial columnar uterus externus, internally with approximately 12 chambers, and an elongated, erect and sinuous spermathecae (Fig. 13F–G).

#### Description.

**Male** (holotype). Total length 1.8. Carapace length 0.6, ovoid; narrowing gradually anteriorly; with purplish pattern pars cephalica flat; fovea not visible (Fig. 13A). Clypeus length 0.7, curved forward. Eyes: PME elongated oval; ALE and PLE rounded. Chelicerae light yellow; promargin with seven teeth, attached to long lamina (Fig. 16A), retromargin without teeth. Sternum light yellow. Endites white yellow suffused. Legs: light purple, formula 1423, total length: I 7.0; II 5.9; III 4.1; IV 6.5. Male palp: palpal femur length 0.4; palpal tibia twice as long as cymbium (Fig. 13C–D); cymbial apophysis long and slightly curved with elongated cuspule at tip, two retrolateral long hairs on projected base, one tarsal organ and three basal setae (Figs 13C, 14B, E–F, 15A); cymbial prolateral extension rounded (Fig. 14A); bulb oval; embolus elongated, flattened at base and with distal area bifid, with short branch notched (Figs 14A–B, 15B–D). Abdomen length 1.3, oval; evenly purplish-green color (Fig. 13A). Six epiandrous spigots, with short base (Fig. 16C).

**Female** (paratype IBSP 176870). Total length 1.9; carapace length 0.74, as in male (Fig. 13B). Pedipalp without claw with conical tip, subdistal trichobothrium and tarsal organ (Figs 16E–F). Clypeus 0.68 diameter. Eyes, chelicerae, sternum, endites (Fig. 16D) and labium as in male. Legs as in male, formula 4123, total length I 6.3; II 4.7; III 3.6 IV4.3. Abdomen length 0.96, globular (Fig. 13B). Colulus triangular, with 9 bristles (Fig. 16B). Internal genitalia with elongated and sinuous spermathecae; long medial columnar uterus externus, longer than spermathecae, with visible chambers. Narrow neck in the columnar uterus externus. Small oval pore-plates on the spermathecae base, with approximately 8–10 glandular ducts (Fig. 13E–F).

#### Distribution.

Recorded exclusively from caves in the Carajás region, state of Pará, northern Brazil (Fig. 19B).

### Ochyrocera charlotte sp. n.

Taxon classificationAnimaliaAraneaeOchyroceratidae

http://zoobank.org/7D7713DC-9B56-4B8F-994C-69299390BCA0

Figures 17 , 20B

#### Types.

Holotype male from Cave N8_0038 (6°10'24"S, 50°8'49"W), 02–29/IV/2015 (IBSP 188897) and paratype female from Cave N1_0247 (6°1'14"S, 50°16'22"W), 03–17/XII/2014 (IBSP 188896), both from Serra Norte, Floresta Nacional de Carajás Parauapebas, Pará, Brazil, Equipe Carste et al.

#### Other material examined.

None.

#### Etymology.

The specific name refers to Charlotte, the spider from the classic “Charlotte’s Web” by E.B. White and a great friend of the pig named Wilbur.

#### Diagnosis.

**Figure 17.**
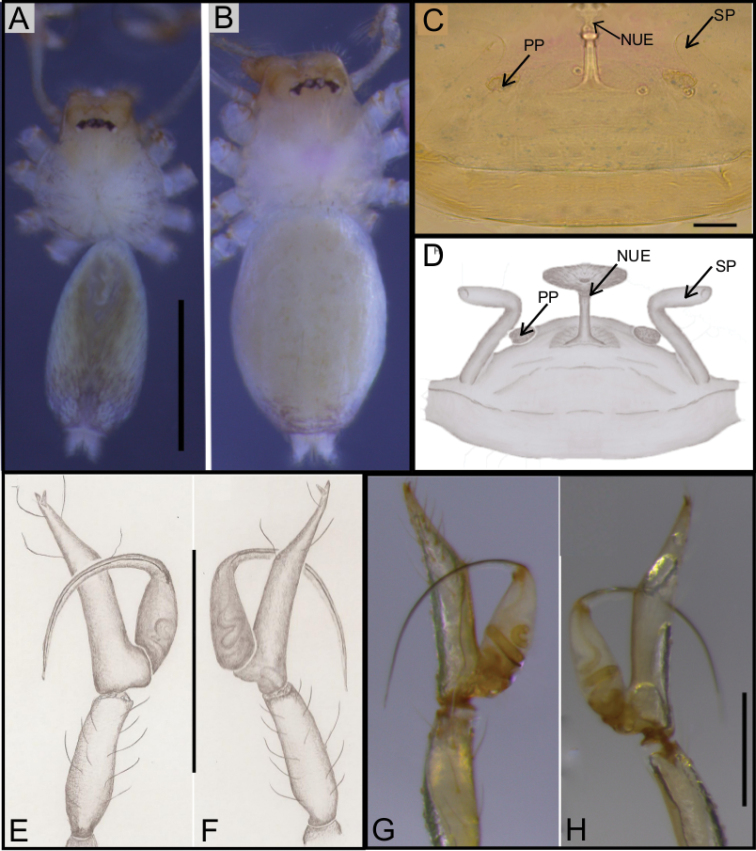
*Ochyrocera charlotte* sp. n., male holotype (**A, E–H**), female paratype, IBSP 188896 (**B, C–F**) **A–B** habitus, dorsal view **C** genitalia, enzyme cleared, dorsal view **D** same, dorsal view **E** same, retrolateral view **F** same, prolateral view **G** left palp, prolateral view **H** same, retrolateral view. Abbreviations: NUE = neck of uterus externus, PP = pore-plate, SP = spermathecae. Scale bars: 0.5 mm (**A, B**); 0.7 mm (**C, D**); 500 µm (**E, F**).

Males and females of *Ochyrocera charlotte* sp. n. resemble those of *O. ungoliant* and *O. viridissima* Brignoli in having a subapical cuspule in the distal area of the cymbium (Fig. 17E−F; Brignoli 1974: fig. 6) but can be distinguished from these species by their yellowish cephalic area and cream body color pattern. Males can be distinguished by their pentagonal cymbium, with cylindrical tegulum (Fig. 17E–H). Females are diagnosed by the genitalia with a very narrow medial columnar uterus externus and an elongated and medially curved spermathecae (Fig. 17C–D).

#### Description.

**Male** (holotype). Total length 2.3. Carapace length 0.7, ovoid; narrowing gradually anteriorly; with yellowish cephalic area, and cream pattern poteriorly, pars cephalica flat (Fig. 17A); fovea not visible. Clypeus length 0.7, curved forward. Eyes: PME elongated oval; ALE and PLE rounded. Chelicerae light yellow, promargin with eight teeth, attached to long lamina; retromargin without teeth. Sternum yellowish. Endites dark yellow. Legs: light yellow; formula 1423; total length: I 7.0; II 5.9; III 4.1; IV 6.5. Male palp: palpal femur length 0.4; palpal tibia narrow, same length as cymbium; cymbial apophysis not curved distally, retrolateral long hair on non-projected base, next to the tarsal organ; with a long hair; with three basal setae; cymbial prolateral extension squared; very coiled spermatic ducts, embolus elongated and flattened (Fig. 17E–H). Abdomen length 1.3, oval; uniformly cream with dorsal light brown band (Fig. 17A). Six epiandrous spigots, with short base.

**Female** (paratype IBSP 188896). Total length: 2.0; carapace length: 0.74; Carapace pattern as in male (Fig. 17B). Pedipalp without claw with conical tip and subdistal trichobothria. Clypeus: 0.68 diameter; Eyes, chelicerae, sternum, endites, and labium as in male. Legs as in male; leg formula 4123, total length: I 6.3; II 4.7; III 3.6 IV4.3. Abdomen length 0.96. Colulus triangular with 8–10 bristles. Internal genitalia with weakly sclerotized, long, curved spermathecae; long medial columnar uterus externus, shorter than spermathecae, with two distal chambers. Narrow neck in the columnar uterus externus. Small oval pore-plates far from the spermathecae base, with approximately 8–10 glandular ducts (Fig. 17C–D).

#### Distribution.

Recorded exclusively from two caves in the Carajás region, state of Pará, northern Brazil (Fig. 20B).

### Ochyrocera ungoliant sp. n.

Taxon classificationAnimaliaAraneaeOchyroceratidae

http://zoobank.org/5FBC7B7B-B21D-4B3B-8921-C1A7659E4AA5

Figures 18 , 20A

#### Types.

Holotype male from Cave N4E_0062 (6°2'1"S, 50°9'12"W), Serra Norte, Floresta Nacional de Carajás, Parauapebas, Pará, Brazil, 24–30/VII/2009, R. Andrade & I. Cizauskas et al. (IBSP 174063). Paratype: female from Cave N4E_0046 (6°2'16"S, 50°9'36"W), Serra Norte, Floresta Nacional de Carajás, Parauapebas, Pará, Brazil, 18/VIII-03/IX/2009, R. Andrade & I. Cizauskas et al. (IBSP 174062).

#### Other material examined.

BRAZIL. Pará: **HYPOGEAN SAMPLES**: Parauapebas, Floresta Nacional de Carajás, Serra Norte, Cave N4E_0046 (6°2'16"S; 50°9'36"W), 1♀, 18/VIII-03/IX/2009 (IBSP 188868); Canaã dos Carajás, Floresta Nacional de Carajás, Serra Sul, Cave S11D-0064 (6°23'31"S, 50°18'48"W), 1♂, 13–30/I/2010, R. Andrade & I. Cizauskas et al. (IBSP 175488); Níquel Vermelho, Cave NV 07 (6°28'41"S, 49°54'20"W), 1♂, 22–28/II/2005, R. Andrade & I. Armoni (IBSP 55367).

#### Etymology.

The specific name in apposition refers to Ungoliant, an evil spider spirit created by J. R. R. Tolkien in the book “The Silmarillion”.

#### Diagnosis.

**Figure 18.**
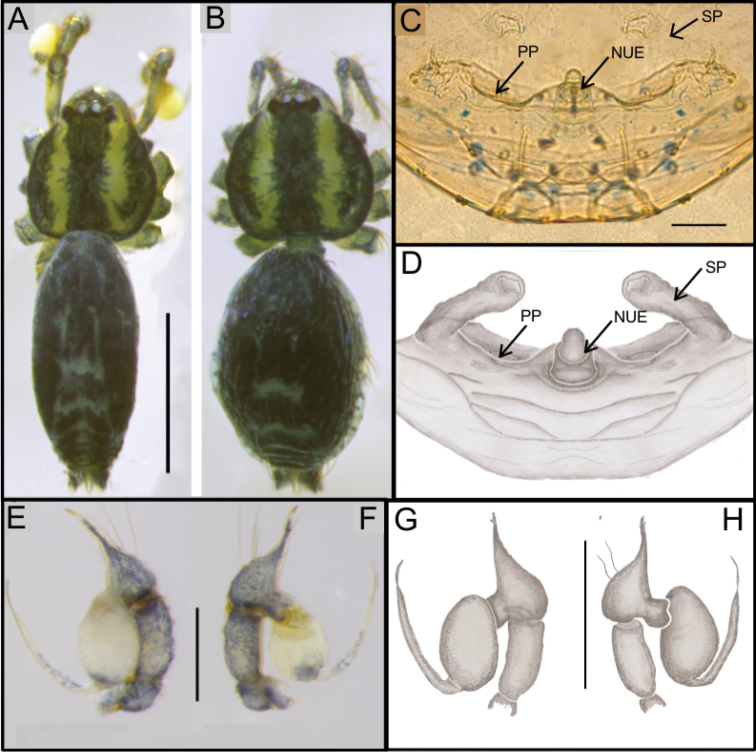
*Ochyrocera ungoliant* sp. n., male holotype (**A, E–H**), female paratype, IBSP 174062 (**B–D**) **A–B** habitus, dorsal view **C** genitalia, enzyme cleared, dorsal view **D** same, dorsal view **E** same, prolateral view **F** same, retrolateral view **G** left palp, prolateral view **H** same, retrolateral view. Abbreviations: NUE = neck of uterus externus, PP = pore-plate, SP = spermathecae. Scale bars: 0.5 mm (**A, B**); 0.7 mm (**C, D**); 500 µm (**E, F**).

Males and females of *Ochyrocera ungoliant* resemble those of *O. charlotte* and *O. viridissima* Brignoli in having a subapical cuspule in the distal area of the cymbium (Fig. 18G−H; Brignoli 1974: fig. 6), but can be distinguished from these species by their intense dark green color pattern and carapace with two longitudinal yellowish-green dorsal bands (Fig. 18A–B). Males can be diagnosed by their short cymbial apophysis with a very narrow tip and embolus with lamellar area in the distal third (Fig. 18E–H); and females by their genitalia with very short medial columnar uterus externus and spermathecae with broad and furrow apex (Fig. 18C–D).

#### Description.

**Male** (holotype). Total length 2.3. Carapace length 0.7, ovoid; narrowing gradually anteriorly; pars cephalica flat; fovea not visible (Fig. 18A). Clypeus length 0.7, curved forward. Eyes: PME elongated oval; ALE and PLE rounded. Chelicerae green; promargin with eight teeth, attached to long lamina; retromargin without teeth. Sternum light green. Endites dark green suffused. Legs: light green; formula 1423; total length: I 7.0; II 5.9; III 4.1; IV 6.5. Male palp: palpal femur length 0.4; palpal tibia not enlarged basally; cymbial apophysis same length as tibiae, with subdistal cuspule at tip, retrolateral long hair on projected base, next to the short tarsal organ; with three basal setae, and cymbial prolateral extension squared; bulb oval; embolus elongated, wide at base and curved forward, with sinuous tip (Fig. 18E–H). Abdomen length 1.3, oval; uniformly purplish green color (Fig. 18A). Six epiandrous spigots, with short base.

**Female** (paratype IBSP 174062). Total length 2.0. Carapace length 0.74. Carapace as in male (Fig. 18B). Pedipalp without claw with conical tip and subdistal trichobothria. Clypeus 0.68 diameter. Eyes, chelicerae, sternum, endites, and labium as in male. Legs as in male, formula 4123, total length I 6.3; II 4.7; III 3.6 IV 4.3. Abdomen length 0.96, with pattern as in male (Fig. 18B). Colulus triangular, with 8–10 bristles. Internal genitalia with weakly sclerotized; short columnar uterus externus, with inconspicuous chambers. Uterus externus ending in a rounded neck in the columnar uterus externus. Small oval pore-plates far from the spermathecae base, with approximately 10–12 glandular ducts (Fig. 18C–D).

**Figure 19.**
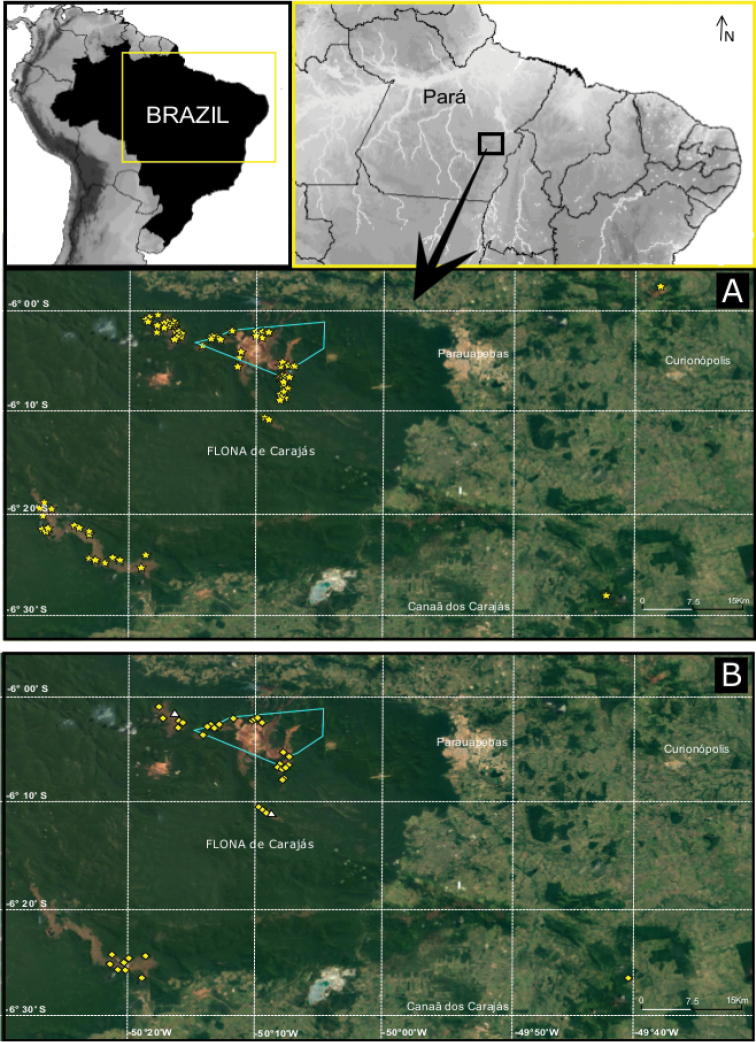
**A** Distribution map of *Ochyrocera varys* sp. n., yellow star **B** *Ochyrocera misspider*, yellow diamond, and *O. aragogue* sp. n., white triangle.

#### Distribution.

**Figure 20.**
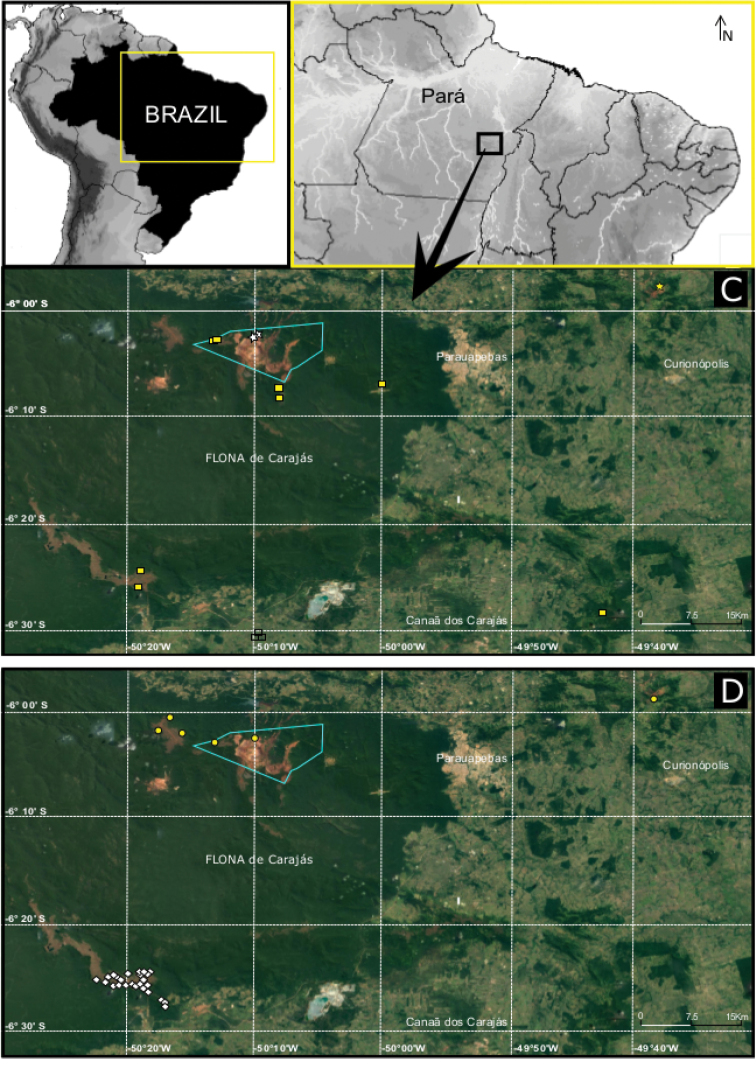
**A** Distribution map of *Ochyrocera laracna* sp. n., yellow square and *O. ungoliant*, white star **B** *Ochyrocera atlachnacha*, white diamond and *O. charlotte* sp. n., yellow circle.

Recorded exclusively from three caves in the Carajás region, state of Pará, northern Brazil (Fig. 20A).

## Discussion

*Relationships*. The inclusion and maintenance of these species in the genus *Ochyrocera* is mainly justified by the male palps following the standard of the type-species, *Ochyrocera arietina* (see Simon 1891: plate XLII, fig. 10): cymbium with a prolateral extension and a distinct distal apophysis, bearing an apical cuspule. Simon (1891, plate XLII, fig. 11) also describes *Ochyrocera quinquivittata* from the same region; however, males of this species have a conical palpal cymbium with a conspicuous retrolateral apophysis.

Since the proposition of the genus, as observed by Hormiga et al. (2007: 16), *Ochyrocera* can be separated into two different groups of species: those with an entire cymbium, with no retrolateral apophysis (proposed here as *arietina* group) and those with an apparently bifid retrolateral apophysis (*quinquivittata* group). Pérez-González et al. (2016) questioned the ambiguous relationship between *Ochyrocera* and *Fageicera* Dumitrescu & Georgescu, 1992, suggesting these genera could be synonymous. Species of *Fageicera* resemble those of the *quinquivittata* group due to the presence of a bifid cymbium, and this characteristic may be a putative synapomorphy for this group. In this case, all *Ochyrocera* species with this feature should be transferred to *Fageicera*. In addition to the three species described by Dumitrescu and Georgescu (1992), species such as *Ochyrocera cachote* Hormiga et al., 2007 and *O. otonga* Dupérré, 2015 could also be included in the genus *Fageicera*. Nevertheless, this can only be solved by an accurate examination of type-species of genera such as *Pandeus* Keyserling, 1891 and *Ceruleocera* Marples, 1955, currently synonyms of *Ochyrocera* (see comments in Pérez-González et al. 2016: 41), and by a cladistic analysis of all species under these generic names.

The aim of this work is not to propose a phylogeny of the genus, but to show that all species herein described have affinities with the *arietina* group. Among the newly described species, *Ochyrocera varys* sp. n., *O. atlachnacha* sp. n., and *O. misspider* seem to be related to the type species, since the male palpal cymbium have distinct distal apophysis, bearing a typical apical cuspule. This character state appears to be the most common among American species (see Dupérre 2015, Pérez-Gonzáles et al. 2016, Valdez-Mondragón 2017). In this scenario, *Ochyrocera misspider* sp. n. may be a sister group of *O. caeruleoamethystina* Lopez & Lopez, 1997, from French Guyana, and *O. thibaudi* Emerit & Lopez from Antilles, since they share the same type of retrolateral projection with long setae on the cymbium (see Lopez and Lopez 1997, fig. 8; Emerit and Lopez 1985, fig. 1A). *Ochyrocera charlotte* and *O. ungoliant* also have a distinct distal apophysis, such as *O. viridissima* Brignoli (see Brignoli 1974: fig.6); however, they have subapical cuspules, a unique characteristic of these three species among those described so far for the Neotropical region.

*Ochyrocera laracna* sp. n. and *O. aragogue* sp. n. form a distinct group of species among those with distal apophysis. In these species, the cymbium of the male palps bears two apical cuspules and this characteristic seems to be exclusive of these two species among the Neotropical *Ochyrocera*.

*Distribution and ecological notes.* In general, the Ochyroceratidae are poorly known in the Neotropical region. Data on the diversity and ecological features of the group are lacking, and most studies are carried out in restricted areas. The group`s known diversity should be larger than it currently is. In this paper, we decribe seven new species of the genus *Ochyrocera* with different distributions and patterns collected in ferruginous caves in the region of Floresta Nacional de Carajás, in the state of Pará, Brazil.

**Figure 21.**
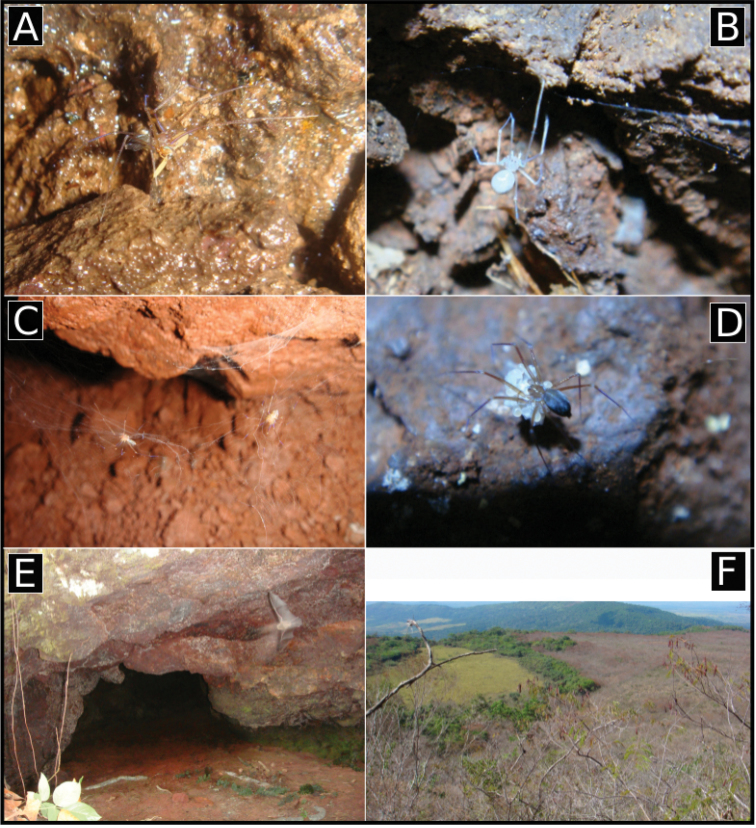
**A** *Ochyrocera varys* sp. n., predating a Diptera **B** *Ochyrocera atlachnacha* sp. n., on the web **C** *Ochyrocera misspider* sp. n., couple in the web **D** *Ochyrocera varys* sp. n., carrying the egg sac **E** Entrance of an iron cave **F** Canga vegetation on rocky outcrop.

The specimens were manually collected inside caves of Floresta Nacional de Carajás, with the aid of brushes and tweezers, and with pitfall and Vulcan traps placed inside and around cave entrances (Piló and Andrade 2007; Cizauskas and Giroti 2011; Bichuette et al. 2015). Collections have been carried out annually in the Carajás region since 2005, usually comprising thirty-day visits with random sampling, aiming at the production of faunistic and environmental reports for the Brazilian environmental protection agency (IBAMA 2017). In general, the cave specimens occupy ground areas near the base of the lateral walls of the caves, where they construct their small webs of refuge (Fig. 21A–F). Spiders collected outside the caves are usually in the shallow litter layer on the ground, building their webs among dead leaves as observed by Jocqué & Dippenaar-Schoeman (2006).

Floresta Nacional de Carajás is part of a conservation unit comprising a large forest island in the southwest of Pará. The area is currently surrounded by pastures, which replaced the original forest (Martins et al. 2012; Campos and Castilho 2012), and comprises 411,949 hectares, covering the municipalities of Parauapebas, Canaã dos Carajás and Água Azul do Norte. The climate type is Montano or Serrano Amazon, with average annual temperatures between 21–22°C. The predominant phytophysiognomy in Floresta Nacional de Carajás is the Equatorial Forest of Terra Firme, with natural clearings such as rupestrian fields or cangas (Ab’Saber 1986).

All species of *Ochyrocera* found in Floresta Nacional de Carajás can be classified as edaphic troglophiles, organisms that can complete their life cycle in the soil, shallow subterranean habitats or in caves (Fig. 21E−F; Sket 2008; Culver and Pipan 2009, Gavish-Regev et al. 2016). During the last five years, we have collected ca. 2000 adult specimens in caves (see examined material above). *Ochyrocera varys* sp. n. (352♂♂, 875 ♀♀) and *O. atlachnacha* sp. n. (131♂♂, 371♀♀) were the most abundant in our collections. Only the species *O. varys* sp. n. and *O. laracna* sp. n. were observed both in caves and epigeal environments, with 9♂♂, 16♀♀ and 45♂♂, 28♀♀ collected, respectively. Besides these species, only *O. misspider* sp. n. exceeded 180 specimens collected in caves (5♂♂, 182♀♀). Other species were represented by ten specimen’s maximum (see examined material).

Floresta Nacional de Carajás has two great ferruginous blooms, Serra Norte (Paruapebas) and Serra Sul (Canaã dos Carajás) (Beisiegel 2006). The species *Ochyrocera charlotte* sp. n. and *O. aragogue* sp. n. were found only in caves from Serra Norte, whereas *O. atlachnacha* sp. n., the second most abundant species, was exclusively found in Serra Sul caves. The other species were sampled in both cave areas (Figs 19–20). Another result of these collections was that five of these species were also found in caves located outside Floresta Nacional de Carajás, with the exception of *Ochyrocera charlotte* sp. n. and *O. atlachnacha* sp. n., which were restricted to the caves of the Floresta Nacional de Carajás (Fig. 20B). Among all species collected, we found no characteristic that infers isolation to the underground environment and these species can be classified as edaphic troglophiles, capable of completing its life cycle in soil, shallow subterranean habitats, or caves.

Most specimens of all the species herein described are located in caves in the Floresta Nacional de Carajás. This area has been an environmental impact target due to the mining process. The effect of this impact in local spider populations has not yet been fully evaluated, especially as large samplings are lacking in other areas, especially those outside caves.

## Supplementary Material

XML Treatment for Ochyrocera

XML Treatment for Ochyrocera varys

XML Treatment for Ochyrocera atlachnacha

XML Treatment for Ochyrocera laracna

XML Treatment for Ochyrocera aragogue

XML Treatment for Ochyrocera misspider

XML Treatment for Ochyrocera charlotte

XML Treatment for Ochyrocera ungoliant
